# Real-time iTRAQ-based proteome profiling revealed the central metabolism involved in nitrogen starvation induced lipid accumulation in microalgae

**DOI:** 10.1038/srep45732

**Published:** 2017-04-05

**Authors:** Vineeta Rai, Muthusivaramapandian Muthuraj, Mayuri N. Gandhi, Debasish Das, Sanjeeva Srivastava

**Affiliations:** 1 Department of Biosciences and Bioengineering, Wadhwani Research Center for Biosciences and Bioengineering, Indian Institute of Technology Bombay, Powai, Mumbai 400076, Maharashtra, India; 2 Department of Biosciences and Bioengineering, Centre for Energy, Indian Institute of Technology Guwahati, Assam 781039, India; 3 Centre for Research in Nanotechnology & Science, Indian Institute of Technology Bombay, Powai, Mumbai 400076, India; 4 DBT PAN IIT Centre for Bioenergy, Indian Institute of Technology Bombay, Mumbai, Powai - 400067, India.

## Abstract

To understand the post-transcriptional molecular mechanisms attributing to oleaginousness in microalgae challenged with nitrogen starvation (N-starvation), the longitudinal proteome dynamics of *Chlorella* sp. FC2 IITG was investigated using multipronged quantitative proteomics and multiple reaction monitoring assays. Physiological data suggested a remarkably enhanced lipid accumulation with concomitant reduction in carbon flux towards carbohydrate, protein and chlorophyll biosynthesis. The proteomics-based investigations identified the down-regulation of enzymes involved in chlorophyll biosynthesis (porphobilinogen deaminase) and photosynthetic carbon fixation (sedoheptulose-1,7 bisphosphate and phosphoribulokinase). Profound up-regulation of hydroxyacyl-ACP dehydrogenase and enoyl-ACP reductase ascertained lipid accumulation. The carbon skeletons to be integrated into lipid precursors were regenerated by glycolysis, β-oxidation and TCA cycle. The enhanced expression of glycolysis and pentose phosphate pathway enzymes indicates heightened energy needs of FC2 cells for the sustenance of N-starvation. FC2 cells strategically reserved nitrogen by incorporating it into the TCA-cycle intermediates to form amino acids; particularly the enzymes involved in the biosynthesis of glutamate, aspartate and arginine were up-regulated. Regulation of arginine, superoxide dismutase, thioredoxin-peroxiredoxin, lipocalin, serine-hydroxymethyltransferase, cysteine synthase, and octanoyltransferase play a critical role in maintaining cellular homeostasis during N-starvation. These findings may provide a rationale for genetic engineering of microalgae, which may enable synchronized biomass and lipid synthesis.

Algae-based biofuels are considered to be emerging, and yet remains promising among the alternate bioenergy resources^1^,^2^. Alterations in inoculum size^3^, growth conditions e.g. light intensity^4^,^5^, temperature^6^,^7^, salinity^8^, oxidative stress^9^, UV irradiation^2^, and nutrient starvation particularly nitrogen^6^,^10^,^11^ induce accumulation of neutral lipid which are further transesterified into biodiesel. However, the biomass is severely impaired. The incompetence to synchronise high cell densities and amass neutral lipids is a major snag in the commercialization of algae-based biodiesel^12^,^13^.

Appropriately several “omics” studies particularly transcriptomics are performed to investigate the N-starvation associated lipid accumulation in *Chlamydomonas reinhardtii*^11^,^14^, *Tisochrysis lutea*^15^, *Ostreococcus tauri*^16^, *Haematococcus pluvialis, Nannochloropsis* sp.^17^, and *Chlorella* sp. Limited proteomic investigations have been performed to understand the underlying molecular mechanisms. First ever report on profiling of *C. reinhardtii* proteome during N-starvation was reported by Longworth and co-workers^11^. Similarly, the proteomes of *Nannochloropsis oceanica*^6^ and *Phaeodactylum tricornutum*^18^ were assessed to emphasize alterations in the cellular and metabolic levels to endure N-starvation. Several studies have considered cross-talk among metabolic networks including remodelling of carbon concentrating mechanism^19^,^20^. Unfortunately till date, the biodiesel derived from microalgae could not be scaled-up to commercial marks. *Chlorella* could be established as an industrial strain of choice, as it is fast growing, may accumulate more than 50–70% lipids/gram of dry weight, its genome manipulation is accessible^21^,^22^, and by large it is fit for human consumption^23^,^24^,^25^. Primarily, after lipid extraction, the dilapidated biomass could be consumed in food industries^26^,^27^. The oleaginous microalga *Chlorella* sp. FC2 IITG (here onwards referred to as FC2) isolated by our group^28^ is a natural isolate having high nutritional content. Such features may open up avenues for its application in food industry; consolidating the applications of FC2 in food and fuel industry may aid to cut-down the biodiesel-production cost^29^,^30^,^31^ and lead to a better environmental sustainability^32^,^33^.

The FC2 cells challenged with N-starvation for 160 h accumulated neutral lipids and displayed reduction in protein, carbohydrate and chlorophyll contents and biomass (dry cell weight) at physiological level (Fig. 1A). Herein, we describe the post-transcriptional responses of the N-starved FC2 cells in its induction phase as a virtue of time (40, 88, and 120 h). The global proteome adjustment was investigated using two high-throughput complementary proteomics platform; DIGE and iTRAQ coupled with electrospray ionization quadrupole time-of-flight (ESI-Q-TOF) mass spectrometry in the discovery phase of the study. A few novel targets were validated using immuno- and multiple reaction monitoring (MRM)-assays (Fig. 1B). Data suggested the temporal regulation of several of the proteins associated with carbon partitioning owing to N-starvation. In future, the understanding of the molecular basis of N-starvation induced lipid accumulation may open-up avenues for industrial application.

## Results

### Effect of N-starvation on the physiology of FC2

**Figure 1:**
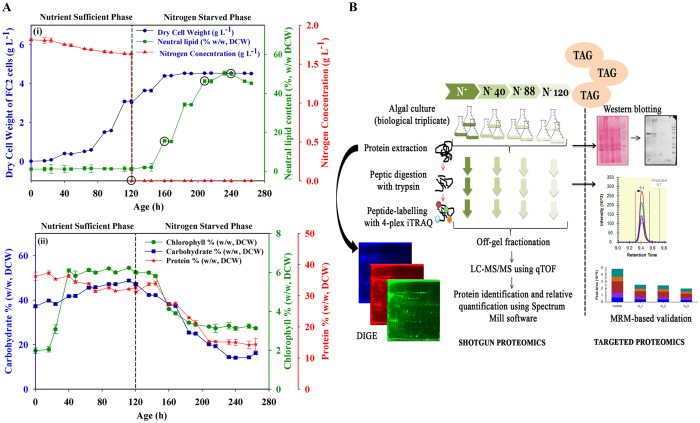
Differential expression studies of FC2 as a function of time till 160 h of N-starvation. (**A**) Physiological studies of FC2 in nitrogen sufficient and starvation conditions. (i) Dynamic profiles of nitrogen utilization, dry cell weight and neutral lipid accumulation; the time-points encircled (0, 40, 88 and 120 h) were selected for proteomics study (ii) comparison of protein, carbohydrate and chlorophyll content. The experiments were conducted in biological triplicate and the data obtained were expressed as mean ± standard error. (**B**) Schematic representation of the experimental strategy used for comparative analysis of differentially expressed FC2 proteome.

A two-stage cultivation strategy was employed (as discussed in methods) to understand the effect of N-starvation on the growth and lipid accumulation of FC2. The nitrogen concentration in the media was maintained at levels not below 1.6 g L^−1^ during the N-sufficient condition whereas under N-starvation stages the concentration was maintained at 0 g L^−1^ as depicted in Fig. 1A(i). Under nutrient sufficient condition the average specific growth rate was 0.053 h^−1^, which gradually decreased during the N-starvation phase. The neutral lipid content (estimated by nile red staining) increased from 1% (w/w, DCW) to 15.48% (w/w, DCW) in the initial 40 h of starvation and reached the maximum of 50.34% (w/w, DCW) by 120 h (Fig. 1A(i)). A concomitant decrease in protein, carbohydrate and chlorophyll contents was observed over the starvation period (Fig. 1A(ii)). Based on the eminent physiological adjustments in FC2 cells to combat N-starvation over the growth period, the time points; 0, 40, 88 and 120 h were selected for the temporal comparative proteomic analysis as encircled in the Fig. 1A(i).

### Identification of differentially expressed FC2 proteins during N-starvation by DIGE analysis

**Figure 2:**
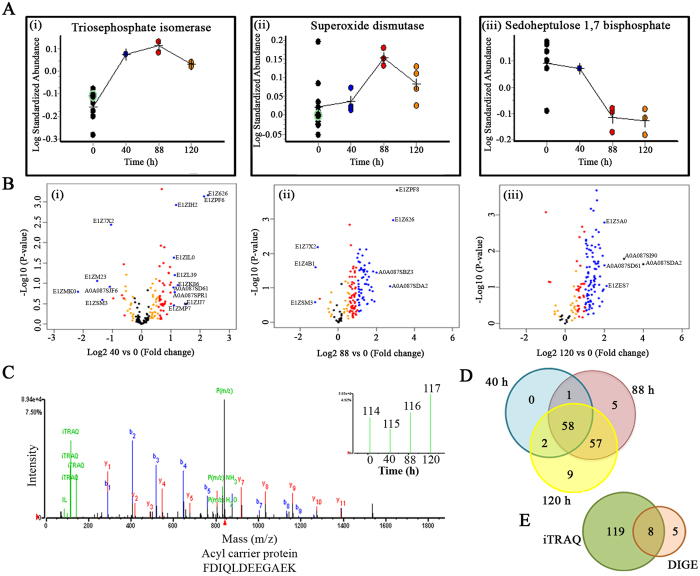
Shotgun proteomics study of FC2 exposed to varied N-starvation conditions. (**A**) Comparative fluorescence intensities of few selected statistically significant (p < 0.05; paired t-test and one-way ANOVA) proteins expressed differentially during N-starvation identified in biological variation analysis (BVA) using DeCyder 2D software; (**B**) Volcano plots showing P values (−log10) versus protein ratio of (log2). Blue > 2 fold change; red > 1.5 fold change, orange > 1.2 fold change and black- no significant change (p-value > 0.05). A few selected differentially abundant proteins are labelled; (**C**) Representative MS/MS spectra of ACP showing higher accumulation with progressive N-starvation time; (**D**) Venn diagram showing the unique and overlapping differentially abundant proteins (p-value ≤ 0.05) in different N-starvation time points.; (**E**) Venn diagram showing the unique and overlapping proteins identified in iTRAQ and DIGE.

DIGE-based comparative proteome analysis of N-sufficient and various N-starvation stages (40, 88 and 120 h) indicated differential expression in multiple protein-levels. Twelve DIGE gels (Fig. S1) were run in different combinations (Table S1) to obtain 68 statistically significant (*p* < 0.05) differential protein spots (31 up-regulated and 37 down-regulated) using DeCyder 2D software (Table S2). The protein spots were marked on the 2DE gel (Fig. S2) excised and subjected to MS and MS/MS analysis to identify 13 non-redundant spots using MASCOT search engine against NCBI nr database (Table S3). Few spots remained unidentified due to the extremely low intensity and inadequate quantity of detectable peptides. Ribose-5-phosphate isomerase (RPI), fructose-1, 6-bisphosphate aldolase (ALDO), unknown protein 18, sedoheptulose-1, 7-biphosphatase (SBP), hypothetical protein CHLNCDRAFT_56187 and triosephosphate isomerase cytoplasmic type (TPI) were identified as multiple spots in DIGE gels, probably due to the presence of multiple isoforms. Among the identified differentially expressed proteins RPI, TPI, SBP, thioredoxin-like protein, alpha-tubulin, reversibly glycosylated protein (RGP), oxygen-evolving enhancer protein, 33 kDa oxygen evolving protein of photosystem II, ALDO, putative 40 S ribosomal protein S3 (partial), malate dehydrogenase (MDH), beta-cyanoalanine synthase/Cysteine synthase (cysK) and actin were found globally in all conditions (0, 40, 88 and 120 h), however, levels of differential regulations (fold-changes) were distinct. Expression trends of some of the proteins are presented in Fig. 2A.

### Altered proteome repertoire of FC2 exposed to N-starvation detected in iTRAQ-based quantitative proteomic analysis

The iTRAQ-based quantitative temporal proteomic analysis in combination with Q-TOF of FC2 cells grown for different N-starvation duration (40, 88 and 120 h) identified around 200 differentially expressed proteins at 1% FDR. Of which, 132 candidates were either with ≥2 unique peptides or present in at least two biological replicates (Table S4). The MS proteomics data have been deposited to the ProteomeXchange Consortium (http://proteomecentral.proteomexchange.org) via the PRIDE partner repository^34^ with the dataset identifier PXD004712. Quantile normalization was employed and a strict confidence score ≥1.3 was used as qualification criteria (Table 1). Volcano plots showing *p* values versus log2 fold change of 40/0, 88/0 and 120/0 h are represented in Fig. 2B. MS/MS spectra of one such peptide, FDIQLDEEGAEK unique to Acyl carrier protein (ACP) with an inset depicting the iTRAQ reporter ion intensities representing different N-starvation time points is shown in Fig. 2C. Comprehensive iTRAQ data analysis revealed a subset of 58 proteins present in all N-starvation time-points, while 5 and 9 different proteins (majorly including protein classes belonging to integral membrane component and carbohydrate metabolism) were expressed exclusively in 88 and 120 h of starvation, respectively. Yet others were involved in adaptation to prolonged N-starvation; interestingly 57 proteins were regulated during the transition from 88 to 120 h of N-starvation (Fig. 2D). A comparison of DIGE and iTRAQ analysis indicated that iTRAQ provides more comprehensive proteome coverage as compared to DIGE. Eight proteins were found to be in common however, five proteins namely thioredoxin-like protein, alpha-tubulin, oxygen-evolving enhancer protein, 33 kDa oxygen evolving protein of photosystem II, and actin were detected exclusively in DIGE analysis, possibly because the 2DE spots were searched against Viridiplantae (Fig. 2E). The proteins displaying differential expressions were clustered on the basis of their biological role (Fig. S3).

### Pathway analysis to map significantly modulated proteins during temporal N-starvation in FC2

**Figure 3:**
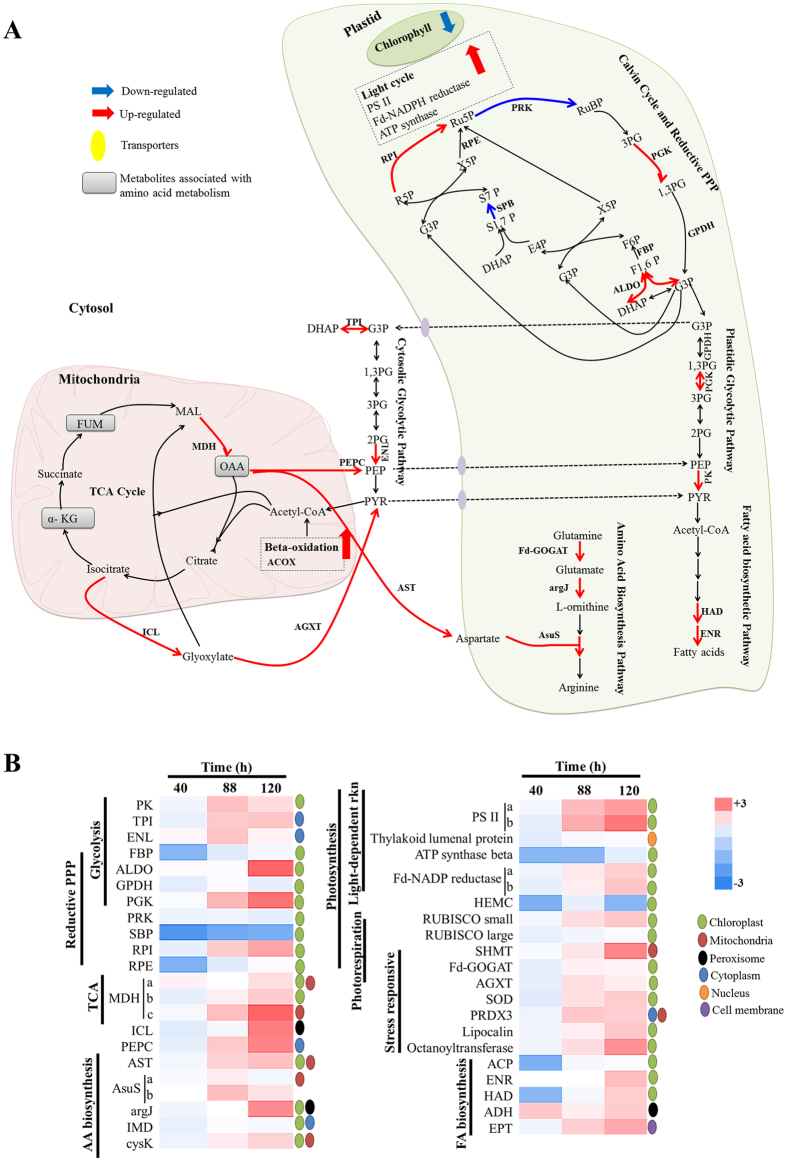
Post-transcriptional regulation of carbon and nitrogen metabolism related to N-starvation-induced lipid accumulation in FC2. (**A**) Regulation of central carbon and nitrogen metabolic pathways related to TAG biosynthesis. The regulatory proteome is indicated by blue (down-regulated) and red (up-regulated) arrows, respectively; (**B**). Heat map illustrating the post-transcriptional dynamics of individual proteins in the central carbon and nitrogen metabolic pathways in response to N availability. RuBP: ribulose-1,5-bisphosphate, 3PG: 3-phosphoglycerate, PGK: phophoglycerate kinase, 1,3PG: 1,3-bisphosphoglycerate, GPDH: glyceraldehyde-3-phosphate dehydrogenase, G3P: glyceraldehyde 3-phosphate, TPI: triosephosphate isomerase, DHAP: dihydroxyacetone phosphate, F1,6P: fructose-1,6-bisphosphate, F6P: fructose-6-phosphate, E4P: erythrose 4-phosphate, X5P: xylulose 5-phosphate, S1,7P: sedoheptulose-1.7-bisphosphate, S7P: sedoheptulose-7-phosphate, R5P: ribose-5-phosphate, RPI: ribose-5-phosphate isomerase, Ru5P: ribulose-5-phosphate, RPE: ribulose-phosphate-3-epimerase, 2PG: 2-phosphoglycerate, ENL: enolase, PEP: phosphoenolpyruvate, PK: pyruvate kinase, PYR: pyruvate, HAD: hydroxyacyl-ACP dehydrogenase, ENR: enoyl-ACP reductase, ALDO: fructose-1,6-bisphosphate aldolase, OAA: oxaloacetic acid, α-KG: α-ketoglutarate, FUM: fumarate, MAL: malate, MDH: malate dehydrogenase, ICL: isocytrate lyase, HEMC: Porphobilinogen deaminase, AsuS: Arginosuccinate synthase, PEPC: phosphoenolpyruvate carboxykinase, AST: aspartate aminotransferase, Fd-GOGAT: ferrodoxin-dependent glutamate synthase, argJ: arginine biosynthesis bi-functional protein, CysK: Cysteine Synthase. Regulation in the proteome are enlisted in Table 1.

To obtain biological meaning at metabolic scale, the differentially expressed FC2 proteins obtained in iTRAQ and DIGE analysis were integrated and highlighted in all possible KEGG (Kyoto Encyclopedia of Genes and Genomes) metabolic pathways using the KEGG Mapper tool. KEGG pathway category enrichment analysis indicated that carbon metabolism including glycolysis, reductive pentose phosphate pathway, TCA cycle, photosynthesis, amino acid assimilation via biosynthesis or protein degradation and fatty acid metabolism were significantly enriched (Fig. 3A, Table 2). The coverage of differentially expressed proteins was assessed by gene ontology terms using Plant-mPLoc (http://www.csbio.sjtu.edu.cn/bioinf/plant-multi/)^35^. Most of the proteins belonged to plastids and mitochondria. Heat maps showing the levels of differential regulation of each proteins belonging to different metabolic process along with their specific spatial location are presented (Fig. 3B).

### Confirmation of the expression levels of a few selected proteins in N-starved FC2 by MRM assay and western blotting

**Figure 4:**
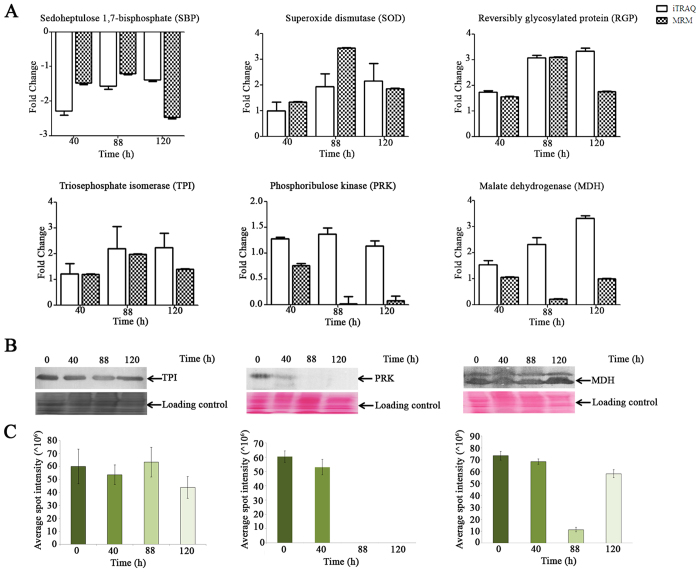
Validation of selected protein showing differential expression in iTRAQ experiments. (**A**) Relative quantification of SBP, SOD, RGP, TPI, PRK and MDH using MRM and comparisons with the iTRAQ data in association with time-dependent N-starvation. Hollow bars represents iTRAQ data, checked bars represents MRM data. (**B**) Western blotting (WB) with three such proteins TPI, PRK and MDH are presented. WB is in concord with the MRM. C: Semi-quantitative estimation of WB bands using iQTL software. The volume intensities obtained were expressed as mean ± standard deviation (n = 3). SBP: sedoheptulose-1,7-bisphosphate, SOD: superoxide dismutase, RGP: reversibly glycosylated protein, TPI: Triosephosphate isomerase, PRK: phosphoribulose kinase, MDH: malate dehydrogenase.

The targeted proteomic analysis was performed using MRM to relatively quantify unique peptides specific for differentially expressed proteins in N-starved FC2 samples (0, 40, 88 and 120 h). MRM transitions were detected, validated and optimized to create a final list of transitions (detailed in Methods). The schematic representation of the steps employed for MRM optimization is shown in Fig. S4. Although aspartate aminotransferase (AST), enolase (ENL), cysK, enoyl-[ACP]-reductase I (ENR) showed significant levels of differential expression in iTRAQ list, no MRM transition could be established (data not shown). MRM assay for six FC2 proteins namely SBP, Superoxide dismutase (SOD), RGP, TPI, MDH, and phosphoribulose kinase (PRK) and bovine serum albumin (BSA; spiked as internal standard) could be optimized and their respective chromatogram is presented in Fig. S4. The score, pI, molecular ion, Q1/Q3 transitions, and optimized CE for each peptide obtained in MRM study are illustrated in Table S5. In total, 139 transitions were detected and established from 25 peptides representing six FC2 proteins and BSA. All the four experimental conditions were screened for all the optimized transitions in biological triplicates. The RSD percentage for BSA was less than 20 for all the runs (Fig. S4), and hence proceeded for further analysis using Skyline v3.5 software. The difference in expression patterns of the proteins obtained in MRM assay was compared to iTRAQ data (Fig. 4A). The expression levels of SBP, SOD, RGP and TPI were in accordance with the iTRAQ data; however, PRK and MDH showed inconsistency (Fig. 4A).

Western blotting for TPI, PRK and MDH was performed. TPI was over-expressed throughout N-starvation stages and the expression was consistent with iTRAQ and MRM. In contrast PRK and MDH expression varied from iTRAQ data; while MRM and western blotting data was consistent (Fig. 4B). PRK was down-regulated upon prolonged N-starvation for 88 and 120 h; while the trend for MDH was up-down-up (Fig. 4B). Semi-quantitative estimation of the expression level of these proteins in the western blot was done by measuring the volume intensity of each band using iQTL software (GE Healthcare Life Sciences) and the average volume intensity (n = 3) was plotted with standard deviation (Fig. 4C).

## Discussion

Switching the fuel source from fossils to sustainable bioenergy resources is the need of the hour. Algae-based biofuels have gained much attention recently owing to their superiority over terrestrial biofuel crop sources. Several proteomics investigation of algae has been performed^4^,^10^,^36^,^37^. Gao and co-workers performed comprehensive comparative genomics, transcriptomics and proteomics analysis of *Chlorella protothecoides* sp. 0710 to determine the oil accumulation mechanisms^36^. The study distinguished the autotrophic and heterotrophic growth conditions using gel-based comparative proteomics followed by LC-MS/MS analysis. Ma and group reported varying inoculum size in a non-model green microalga *Chlorella sorokiniana* greatly affects cell density, an essential criteria for industrial production of biodiesel from microalgae. The proteins participating in photosynthesis (light reaction) and Calvin cycle (carbon reaction pathway) displayed highest levels of differential expression under inoculum size of 1 × 10^6^ cells mL^−1^, and lowest under 1 × 10^7^ cells mL^−1^ 4. Guarnieri *et al*.^37^ investigated the global proteome profile of N-starved *Chlorella vulgaris*; briefly two different conditions (N-sufficient and N-starved) were considered using gel-based liquid chromatography-mass spectrometry (GeLC/MS). The data indicated enhanced fatty acid and triacylglycerol biosynthetic machinery under N-depletion condition. However, the proteins identified in the study remained unvalidated^37^.

The present study was thus undertaken to identify the proteins expressed differentially in longitudinal manner in a non-model oleaginous green microalga *Chlorella* sp. FC2 IITG. Nitrogen and phosphate starvation are identified as the two major triggering factor for neutral lipid accumulation in FC2 cells; however N-starvation resulted in rapid changeover in neutral lipid content from 1% to 54.4% (w/w, DCW)^28^. The time point 0 h designates the N-sufficient condition while other three time points viz. 40, 88 and 120 h were taken into account to get a comprehensive insight into the N-starvation induced lipid accumulation pathways. A sharp increase in the neutral lipid content was observed post 40 h followed by an exponential increase in neutral lipid content for up to 88 h, which subsequently dropped following 120 h of starvation (Fig. 1A). These time points thus represent the critical stages for neutral lipid accumulation and hence were selected for proteomic analysis. The preferential utilization of intracellular nitrogen attributed to the constant growth in the N-deprived FC2 cells up until the initial 40 h of starvation^38^. Advanced stages of N-starvation attributed to drastic drop-down in growth rate with concomitant elevation in neutral lipid content (15.48% to 50.34% w/w, DCW). Indeed, N-starvation tends to shut down housekeeping functions; the same is imitated in the present study where protein, carbohydrate and chlorophyll contents were progressively down-regulated in FC2 with prolonged N-starvation duration. The global protein expressions of FC2 as a function of time under N-starvation conditions were evaluated by two complementary proteomics technique; iTRAQ and DIGE, with three biological replicates for each time-point. The iTRAQ data thus obtained was quantile normalized. Furthermore, the time-resolved proteomes were validated by MRM of 6 selected proteins and western blotting for 3 such proteins (Fig. 1). The expression patterns of PRK and MDH determined using western blotting and MRM were in sink, but showed inconsistency with iTRAQ data (Fig. 4). The reason for this discrepancy might be the low abundance of peptides, smaller sample size, and complicated experimental procedures and data analysis lacking suitable internal standards^39^,^40^. Despite several disadvantages, iTRAQ is the most amenable technique to orthogonal separation due to multiplexing that ensures its value for many analysis schemes and the reduction of costly LC–MS runtime^41^,^42^,^43^. The targeted proteomics based on MRM has emerged as a technology to complement the discovery capabilities and overcome the technical pitfalls, such as incomplete protein extraction, proteolysis, and artifactual protein modifications^44^.

Comparative temporal proteomic analysis of FC2 cells indicated regulation of diverse protein classes under the following sub-classes: (a) N-assimilation, amino acid biosynthesis and protein degradation; (b) photosynthesis; (c) energy pathways; (d) fatty acid metabolism; and (e) stress-responsive mechanisms. The discussion hereafter focuses on the specific proteins under these sub-headings identified in our study and their associated role.

### N assimilation, amino acid biosynthesis, and protein degradation

Global proteome re-adjustment in terms of both amino acid biosynthesis and protein degradation (involving proteasomes) pathways was observed in FC2 cells as a feedback mechanism to N-starvation. Overall, the enzymes involved in the biosynthesis of glutamate, aspartate and arginine were elevated. Plastidal ferrodoxin-dependent glutamate synthase (Fd-GOGAT) was 1.5 folds up-regulated following 88 h of N-starvation, suggesting glutamate accumulation in FC2 cells. The GS-GOGAT pathway although energetically expensive, is often triggered during low N- concentrations^45^,^46^. In parallel, the elevated levels of MDH (TCA cycle) and isocitrate lyase (ICL; glyoxylate cycle), suggests enhanced oxaloacetic acid (OAA) accumulation in FC2 cells. Moreover, OAA may be subsequently transaminated to aspartate using the Aspartate aminotransferase (AAT), which again was up-regulated by more than 2 folds in the advanced N-starvation stages. Two of the arginine biosynthetic enzymes namely Arginosuccinate synthase (AsuS) and Arginine biosynthesis bi-functional protein (ArgJ) were up-regulated at 88 and 120 h of N-starvation (Fig. 3, Table 1). Previous studies displayed a positive correlation of transcription factor bHLH6 with AsuS in *Chlamydomonas* during N-starvation^47^. Arginine accumulation is reported during N-deprivation conditions; arginine catabolism assists in mobilizing the stored nitrogen based on the nutritional status of the cells. Besides, arginine plays role in augmenting stress-responsive mechanisms thereby reducing the overall effect of oxidative and other abiotic stresses as reported in *Arabidopsis thaliana*^48^. Arginine accumulation was also mirrored in our parallel metabolomics investigation (data unpublished). The accumulation of arginine, having highest nitrogen to carbon ratio, could be a strategy adopted by the algal cell to store organic nitrogen and combat abiotic stress.

Simultaneously, degradation of proteins using various catabolic enzymes including peptidase, proteasomes and ubiquitin (Table 1) augmented the recycling of nitrogen and several TCA cycle intermediates. These findings are consistent with the transcriptomics and metabolomics^49^, and label-free proteomics^50^ data in *P. tricornutum* (model diatom) following N-starvation. Our data clearly indicates a tight regulation of several enzymes associated with carbon and nitrogen metabolism in response to N-starvation.

### Photosynthesis

N-starvation is often linked to reduction in photosynthetic efficiency, primarily due to chlorosis. Porphobilinogen deaminase, involved in chlorophyll biosynthetic process, was down-regulated by 1.3 folds during initial N-starvation phase (40 h) and corresponds well to the physiology (Fig. 1A). The decline in chlorophyll levels during N-starvation is often associated with rapid cessation in its synthesis and dilution by cellular growth rather than its degradation as reported in *C. reinhardtii*^51^. Interestingly, several of the photosynthetic proteins including photosystem II assembly, E1ZPZ7, E1ZQR2 (KEGG: PSII oxygen evolving enhancer protein 1 and 2; psbp), ferredoxin-NADP reductase, E1ZFB3 (thylakoid lumenal protein), and cytochrome 3 were up-regulated during the process. It has been already shown that the novel isolate FC2 derives energy and carbon for *de novo* TAG synthesis from photosynthesis during N-starvation^19^, although photosynthetic yields are compromised due to reduced chlorophyll content^52^, and photosynthetic carbon fixation^53^. The later is primarily due to curbed regeneration of Ribulose-1,5-bisphosphate carboxylase/oxygenase (RuBisCO)^54^; reduced expressions of PRK and SBP is perhaps the rate limiting step although other photosynthetic related enzymes are up-regulated. Elevated levels of some of these photosynthetic proteins is in corroboration with the previously described plant omics study; for instance psbp was up-regulated in two different maize cultivars grown in low N-conditions^55^; possibly released as a degradation product of oxygen evolving complex proteins that assist FC2 cells in adapting to the adverse conditions^56^.

### Energy pathways

The enzymes ferredoxin-NADP reductase and that of PSII are involved in photophosphorylation that fulfils the energy requirements of the cell. However, under unfavourable conditions the energy is re-directed towards lipid-accumulation which serves as energy-reserve for the cell over the prolonged stress durations^57^. The increased energy requirements to synthesise high-energy compounds are attained by glycolysis, TCA cycle and non-oxidative pentose phosphate pathway (PPP). Likewise, FC2 cells displayed elevated energy-metabolism activities with significant coverage of the proteins linked to glycolysis, TCA cycle and non-oxidative pentose phosphate pathways (PPP). The non-oxidative PPP are primarily involved in the inter-conversion of sugars that can re-enter glycolysis or oxidative PPP for generation of reducing equivalents (NADPH). These reductants, apart from their role in maintaining redox (particularly overcoming oxidative stress), also find function in supporting *de novo* fatty acid biosynthesis^58^,^59^ and N-assimilation^20^. The synergistic actions of PPP and glycolytic pathways using a different combination of their respective enzyme sets provide cells with flexibility to modulate energy levels, reducing power or a combination of these functions^60^. The involvement of glycolytic enzymes namely GPDH, ALDO, TPI and PK has been reported to be the major regulators for lipid induction in oleaginous *Scenedesmus dimorphus, S. quadricauda* and *Mucor circinelloides*^61^,^62^. Co-ordinated expression of the enzymes during N-starvation redirects the carbon-flux from carbohydrate towards neutral lipid biosynthesis via pyruvate, which is the key precursor for acetyl CoA. Likewise, ribose-5-phosphate isomerase (RPI) and ribulose-phosphate-3-epimerase (RPE) functions in sink to supply the carbon and limiting NADH required for lipid biosynthesis. Transcriptomic analysis of N-deprived *Neochloris oleoabundans* revealed over-expression of PPP^63^. In the present study GPDH, PGK, ENL, Fructose-1,6-bisphosphatase (FBP), ALDO, TPI, and PK belonging to glycolytic pathway, and RPI and RPE from non-oxidative PPP work in a cordial manner to generate NADPH and pyruvate that may be converted to ATP (Fig. S5), and fulfil the energy needs of FC2 cells for sustenance of N-starvation. Furthermore, up-regulation of MDH and phosphoenolpyruvate carboxykinase (PEPC) suggests conversion of malate to OAA to phosphoenolpyruvate (PEP), which is subsequently converted to pyruvate via PK. The pyruvate can then enter the TCA cycle and contribute to the production of mitochondrial citrate, which can then feed into the *de novo* fatty acid synthesis upon its export to the cytoplasm^64^. Our data is consistent with the enriched gluconeogenesis transcripts in a starchless mutant of *C. reinhardtii* grown in N-starvation conditions^14^. The FC2 cells ascertained that the PEP is not a limiting factor for ATP and pyruvate generation by elevating the levels of a complementary glycolytic enzyme; ENL. PEP may serve as a precursor molecule for isoprenoid and glycerolipid biosynthesis through pyruvate and acetyl-CoA^65^. Interestingly, the abundance of ENL and PK is in accordance with the temporal lipid accumulation in N-starved FC2 cells as confirmed by nile red staining. An elevated level of ENL is also reported in N-starved rice^66^ and Arabidopsis^67^.

### Proteins associated with fatty acid metabolism

Earlier findings from our group and others have highlighted the complex links between lipid accumulation and N-starvation in microalgae. Transcriptomics study in *Nannochloropsis oceanica* IMET 1 revealed up-regulation of Acyl carrier protein (ACP) by more than 2 folds, while the levels of 3-hydroxyacyl-[ACP]-dehydratase (HAD) and ENR were down-regulated at 48 h under N-deprived conditions^68^. Contrarily in the present study, gradual accumulation of several fatty acid biosynthesis and storage proteins was observed with progressive N-starvation duration. Several components of fatty acid synthase (FAS) including E1Z5W8 (ACP), E1Z8J0 (HAD), and E1Z2Y2 (ENR) were significantly accumulated following 88 h of N-starvation (Table 1). In algae, ACP tethers the growing fatty acid chain as it goes through the elongation step. Dehydratase and reductase catalyzes the third and fourth step of fatty acid elongation, wherein enoyl-ACP losses one molecule of water and is reduced to fully saturated acyl-ACP. Additionally, the involvement of two of the enzymes E1Z2Y2 (ENR) and E1Z8J0 (HAD) in biotin metabolism is highlighted in the KEGG pathway. Regulation of biotin metabolism is correlated with lipid accumulation in *C. reinhardtii*^11^.

The significant upsurge in the fatty acid accumulation during N-starvation may arise either due to (a) the accelerated partitioning of new-photosynthetically fixed carbon through glycolysis, which produces pyruvate from glucose while generating the high-energy compounds ATP and NADH^69^, or (b) the recycling of carbon-molecules into the precursors for lipid biosynthesis.

- In vascular plants and algae, glycolysis occurs in both plastids and the cytosol. However, in the present study, most of the differentially expressed glycolysis-associated proteins were plastid-bound, except for TPI and ENL (present in the cytosol) (Fig. 3). This suggests that plastids possess the enzymatic machinery of the payoff phase of the glycolysis pathway (from G3P to pyruvate) along with plastidic ALDO that converts F1, 6P to G3P and thus the plastid glycolysis pathway generates pyruvate in N-starved condition, which regulate the elongation of both long-chain saturated and unsaturated fatty acids. This observation is in sync with the transcriptomics studies on N-starved *Nannochloropsis oceanica* IMET 1 by Li *et al*.^68^.
- Interestingly, we observed perpetual up-regulation of E1ZIL0 (KEGG: acyl-CoA dehydrogenase activity), a component of complex I of oxidative phosphorylation which suggests that knock out of this enzyme could be detrimental to the organism^70^. E1ZIL0 is also involved in β-oxidation pathway; transcript analysis of *N. oceanica* IMET 1 revealed concomitant up-regulation of several components of β-oxidation pathways in response to N-deprivation^68^. Besides, carbon skeletons to be integrated into the neutral lipids may be salvaged from the degradation of membrane-bound glycerolipids via β-oxidation, suggesting that these enzymes could be sensible targets for maximizing neutral lipid biosynthesis. Knock-out of several lipases involved in β-oxidation pathway has been reported to result in synchronized growth and lipid accumulation^71^.

Present study thus highlights the fact that up-regulation in TCA cycle coupled with increased fatty acid degradation in the mitochondria may enhance recycling of carbon skeleton for neutral lipid accumulation under N-starvation. Mitochondria thus serve as an auxiliary organelle for bulk fatty acid biosynthesis, in a fashion similar to vascular plants^72^. Our data correlates the previously reported transcriptomics studies with the proteome regulation during N-starvation. Notably, ACCase, a key enzyme catalyzing the irreversible step in fatty acid biosynthesis is not expressed differentially in our study. This may be due to the iTRAQ method employed for the comparative proteomics analysis, which apply differences throughout the measurements^73^,^74^,^75^. Suggesting, the difference in the expression level of ACCase in the test and control sample is not significant. Findings from our proteomics study and transcript analysis by Li *et al*.^68^ well explains the failed attempts to enhance lipid content in diatom *Cyclotella cryptica* and *Navicula saprophila*^76^,^77^ and plants^78^ by overexpressing ACCase. Unfortunately, even up to two to three-fold elevated ACCase activity in the transformed algae did not led to any enhancement of lipid production^79^.

### Proteins in handling cellular stress

N-starvation is characterized by oxidative stress in green alga *Chlorella sorokiniana* C3 and *Scenedesmus* sp., and by oxidative stress induced lipid accumulation in *Dunaliella salina*. In the present study, over-production of three stress-responsive enzymes namely SOD, E1ZG17 (KEGG: thioredoxin peroxiredoxin activity) and E1ZCK9 (KEGG: Chloroplastic lipocalin) are involved in the oxidative stress alleviation of FC2 during N-starvation. SOD (E1Z580) catalyzes dismutation of superoxide (O^2−^) into molecular oxygen and hydrogen peroxide, thus contributing to the tolerance towards ROS damage caused by N-starvation conditions. E1ZG17 has a proven anti-oxidant role. Thioredoxin peroxidases from *Synechocystis* sp. PCC 6803 is capable of reducing H_2_O_2_ and its activities are coupled to the photosynthetic electron transport system^80^. The homolog of E1ZCK9 in *Arabidopsis* was reported to be accumulated during stress-conditions and has an evident role in the protection of thylakoidal membrane lipids against ROS^81^. Besides, the involvement of photorespiration to combat the redox stress during N-starvation condition cannot be ruled out. Correspondingly in the present study, four of the enzymes involved in photorespiration pathways namely; Serine hydroxymethyltransferase (SHMT), RuBisCO, Fd-GOGAT and Serine-glyoxylate aminotransferase showed up-regulation with the progression of N-starvation duration. SHMT has been known previously for armoring abiotic stress-triggered cell damage in *Arabidopsis*^82^. Likewise, up-regulation of SHMT during later stages of N-starvation may aid in balancing the cellular redox in N-starved FC2 cells. SHMT reversibly catalyze glycine to serine, which in turn, serves as a precursor for cysteine biosynthesis via cysteine synthase^83^. Cysteine synthase levels were expressed co-ordinately to SHMT, affirming that the cysteine levels are not a limiting factor for glutathione synthesis activity (component of redox homeostasis and detoxification machinery) under N-starved conditions in FC2. On the other hand, cysteine is a limiting factor for glutathione synthesis under N+ conditions. Additionally, enhanced accumulation of Octanoyltransferase, involved in the biosynthesis of lipoic acid has a prominent role in ROS scavenging. Therefore, we hypothesize that the levels of SOD, thioredoxin peroxiredoxin activity, chloroplastic lipocalin, SHMT, cysteine synthase, and octanoyltransferase play a critical role in maintaining cellular homeostasis during N-starvation.

To the best of our knowledge, this is the pioneering systemic study of its kind, where the temporal proteomics analysis of a novel green oleaginous algae *Chlorella* sp. FC2 IITG at lipid induction phase is performed. Overall, the present knowledge laid the background of the post-transcriptional metabolic networks involved in N-starvation linked lipid induction in microalgae. Many of the proteins viz. SBP, PRK (involved in photosynthetic carbon fixation) identified in our study may serve as potential targets for strain improvement (i.e. synchronous growth and lipid accumulation), and pave way for economically viable and sustainable algal-based biofuel. PRK is the rate limiting step in Calvin cycle and down-regulation of this enzyme reduces the regeneration of RuBP, suggesting that enhancing its expression during stress may improve the photosynthetic yields^84^. Interestingly, increased levels of β-oxidation pathway enzymes may function in either way; which may aid in carbon-recycling for lipid biosynthesis or be involved in catabolism of neutral lipids. Thus, selective knock-down of the enzymes may synchronize biomass and lipid accumulation^71^. The impact of SBP in enhancing the plant growth has already been reported by several researchers^85^,^86^. SBP activity is modulated by the environmental factors^87^,^88^,^89^,^90^. The findings is well justified by the fact that transgenic rice over-expressing SBP did not show any significant change in biomass when grown at ambient conditions while enhanced biomass and photosynthesis was recorded when grown under abiotic stress conditions, particularly high salt^89^ and temperature stresses^90^. This is the first-ever report of the MRM-based targeted validation at proteome-level in any algal species, and may be expanded to other microalgae. Although the shotgun data is validated by the western blotting and MRM assays, a more direct proof concerning the role of these proteins towards N-starvation induced lipid accumulation could be reinforced by genetic manipulation of algae for further strain improvement.

## Methods

### Microalgae, growth media and culture conditions

A novel freshwater indigenous microalga FC2, isolated from a North-Eastern part of India^28^ was cultured under a photoautotrophic condition in a slightly modified BG11 medium^91^. Seed cultures (100 mL) were grown in Erlenmeyer flask and incubated at 28 °C, 150 rpm, light intensity 20 μE m^−2^ s^−1^ with a light: dark cycle of 16:8 h till absorbance (*A*_*690*_) of 1.0 was reached in an orbital incubator shaker (Multitron-Pro, Infors HT, Switzerland). 1% (v/v) of the seed culture was used as inoculum for a pilot study conducted in a 5.0 L automated bioreactor (Biostat B plus, Sartorius, Gettingen, Germany) containing 4.0 L of BG11 medium. The reactor conditions were agitator speed of 400 rpm, aeration 1 vvm with 1% (v/v) CO_2_, light intensity 250 μE m^−2^ s^−1^ with a light: dark cycle of 16:8 h, temperature 28 °C and pH 7.4. Samples were collected at regular intervals to assess the growth dynamics, substrate utilization profiles and biochemical composition of the cells.

Two-stage cultivation strategy was employed to understand the effect of N- starvation on growth and lipid accumulation of FC2 cells. In the first stage, FC2 cells were grown under a photoautotrophic condition in the nutrient sufficient BG11 medium to obtain biomass concentration of 3 g L^−1^ (equivalent to absorbance 13.0 at 690 nm). The nutrient sufficient condition was maintained by intermittent feeding of the necessary limiting nutrients when their concentration reduces more than 10% from their optimal concentrations. Once the desired absorbance was reached, FC2 cells were harvested aseptically via centrifugation at 8,000× *g* for 10 minutes at 4 °C and washed with BG11 media devoid of urea. FC2 cells were then re-suspended in BG11 media devoid of urea to grow cell under N-deprived condition. All the other cultivation parameters were kept constant.

### Analysis of growth, biomass composition, and substrate utilization profiles

An equal volume of samples was harvested by centrifugation at 8,000× *g* for 10 minutes at 4 °C for analyzing growth and biomass composition. The biomass was determined as a measure of cell absorbance at 690 nm (A_690_) with a UV-visible spectrophotometer (Cary 50, Varian, Australia) and were expressed in terms of dry cell weight (DCW) using the correlation equation 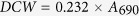 for N-sufficient growth conditions and 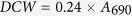 for N-deprived condition^91^. The utilization profile of the nitrogen source (urea) was obtained employing previously reported method^91^. Total carbohydrate in FC2 cells was estimated using phenol-sulphuric acid method proposed by Dubois *et al*.^92^ using glucose as standard. Total chlorophyll was expressed as a measure of chlorophyll *a* and *b*. Chlorophyll was extracted in 100% methanol, chlorophyll *a* and *b* were measured in a UV-visible spectrophotometer using protocol by Pruvost *et al*.^93^, and the amount was calculated using the empirical equation designed by Ritchie *et al*.^94^. Intracellular neutral lipid content was determined by staining FC2 cells with 4 μg mL^−1^ Nile-red dissolved in 25% dimethyl sulfoxide (DMSO) and fluorescence was measured in a spectrofluorometer (Fluoromax 3, Horiba, USA) with excitation at 480 nm and emission in the region 550–650 nm. Triolein (Supelco, USA) was used as the standard for Nile-red based neutral lipid estimation^28^. All the experiments were conducted in biological triplicate and the data is presented as a mean ± standard error.

### Protein extraction, quantification and 2DE clean-up

Total protein was extracted from FC2 cells grown for 40 h, 88 h and 120 h following N-starvation and 0 h (control) using TRIzol reagent following standard protocol with slight modification^95^. Briefly, the algal pellet was incubated in PBS (pH 7.4) containing lysozyme (1 mg mL^−1^) and protease inhibitor cocktail at ambient temperature for 3 h, followed by sonication for 10 min (30 s ON and 30 s OFF cycle) and centrifuged at 12,000× *g* for 15 min. The supernatant was mixed with equal volume of TRIzol reagent and processed to obtain the protein precipitates in acetone. Protein pellets were dissolved in rehydration buffer containing 8 M urea, 2 M thiourea, 2–4% CHAPS, 40 mM 13 DTT and 0.002% bromophenol blue. Algal proteins contain several secondary metabolites, salt, and pigments, so 2DE clean-up was performed using the commercially available kit from GE healthcare. Protein concentrations were determined using QuickStart Bradford reagent (BioRad, USA) and quality was checked on 12% SDS gel.

### DIGE, image acquisition and software analysis

Twelve DIGE gels in different combinations of protein sample types (0 h, 40 h, 88 h and 120 h) were run in order to determine differential protein expressions upon N-starvation (Table S1). Protein extracted from FC2 cells grown in N-sufficient condition (0 h) was used as control, while mixture of protein containing equal amount of proteins from 0 h, 40 h, 88 h and 120 h post N-starvation grew FC2 cultures was used as internal standard, and proteins obtained from different N-starved conditions (40 h, 88 h and 120 h) was used as treatment conditions. Briefly, Cy3 and Cy5 were used to label control and treatments while internal standards were labeled with Cy2 according to the manufacturer’s instructions (GE healthcare). DIGE experiments were performed in replicates of four and dyes were swapped to avoid any type label biases (Fig. S1). Image acquisition and data analysis were performed as per previously described protocol^96^. Differential in-gel analysis (DIA) and biological variation analysis (BVA) of DeCyder 2D software v7.0 (GE Healthcare) was used for comparing control and test sets. Statistical significance of the average ratio of expressions was analyzed by Student’s t-test and ANOVA (p < 0.05).

### In-gel digestion, LC-MS/MS analysis and protein identification

Statistically significant (p < 0.05) proteins showing differential expression profile in DIGE analysis were spotted on 2DE gels and excised. Subsequently, in-gel digestion and enrichment of digested peptides using Zip-Tip C18 pipette tips (Millipore, USA) were performed using previously described protocol^97^. MS and MS/MS was determined using 1260 Infinity HPLC-nano-chip linked to Agilent 6550 iFunnel Q-TOF instrument (Agilent technology, USA) equipped with a Polaris C18A chip (150 mm 3 0.075 mm) with 160 nL trap column. For elution of the peptides from the analytical column, a step-wise gradient of 3–35% ACN for 70 min, 45% ACN at 75 min, 95% ACN at 85 min was used at a flow rate of 2.0 μL min^−1^ for the capillary pump and 0.3 μL min^−1^ for the nano pump. Protein identification was carried out by MS/MS ion search using MASCOT version 2.1 against the NCBI non-redundant database (last updated January, 2015) specifying the following settings; taxonomy: Viridiplantae, trypsin digestion with one missed cleavage, fixed modifications: carbamidomethylation of cysteine residues, variable modifications: oxidation of methionine residues, mass tolerance 75 ppm for MS and 0.4 Da for MS/MS. Identified proteins having at least two unique matched peptides are reported.

### In-solution digestion, iTRAQ labeling and OFF-GEL fractionation

Proteins in rehydration buffer (described earlier) were exchanged to 0.5 M TEAB buffer (compatible for iTRAQ labeling) using Amicon Ultra 0.5 mL centrifugal 3 kDa filters (Millipore, Watford, UK). Following buffer-exchange protein concentrations were determined using QuickStart Bradford reagent (BioRad, USA) and quality was checked on a 12% SDS gel. In-solution digestion of respective protein samples (100 μg each); control (0 h) and treatments (40 h, 88 h, and 120 h) were performed using Trypsin (Trypsin Gold, mass spectrometry grade, Promega, Madison, WI, USA) at a ratio 1: 30 (trypsin: protein), following the manufacturer’s instructions. Four-plex iTRAQ labeling kit (AB Sciex UK Limited, UK) having labels 114, 115, 116 and 117 was used to label trypsin-digested peptides of 0, 40, 88 and 120 h FC2 samples respectively, following the manufacturer’s instructions. All the labeled peptides were pooled and proceeded for OFFGEL fractionation using a 3100 OFFGEL Fractionator (Agilent Technologies, Santa Clara, CA) with high resolution (pH 3–10, 24 cm) IPG strips. Fractions were collected and enriched using Zip-Tip C18 pipette tips (Millipore, USA).

### LC-MS/MS based protein identification database search and quantitation

MS and MS/MS run of OFFGEL fractionated labeled peptide samples were performed following the above-mentioned protocol. Chip-Cube controlled by the Mass hunter acquisition software was set to perform data acquisition in a positive ion mode. MS was scanned from 300–3000 and MS/MS from 50–3000. The instrument was operated in a data-dependent manner using Auto MS/MS, selecting max 15 precursors with intensity over 1000 for each cycle. MS/MS was done with a gas pressure of 2 × 10^−2^ bar in the collision cell. Data files (in.d format) were processed by Spectrum Mill Protein Identification software (Agilent Technologies, USA). The Paragon algorithm was used as the default method for search with trypsin as a digesting agent with up to two allowed miss cleavages. Protein identification was executed against the *uniprot_Chlorella sp.* (dated 30^th^ April 2016; containing 9831 sequences for *C. variabilis* and 7001 sequences for *C. protothecoides*). Data was extracted between MH+ 600 and 4000, precursor mass tolerance 20 ppm and fragment mass error tolerance 50 ppm. Only peptides identified with confidence interval (C.I.) values above 95% were used for protein identification and quantification. The iTRAQ report peak areas (RPAs) corresponding to quantification ions m/z 114–117 were extracted from the raw spectra and corrected for isotopic carryover using GPS Explorer. A decoy database search was used for calculation of the false discovery rate (FDR) and a cut-off of 1% was used to report identifications.

### Protein networks and Functional analysis

Pathway reconstruction of the differentially regulated FC2 proteins (p < 0.05) identified during the progression of N-starvation period was achieved using the KEGG PATHWAY tool available from the Kyoto Encyclopedia of Genes and Genomes (http://www.genome.jp/kegg/tool/map_pathway2.html). The list of UniProt Accession IDs was uploaded and matched against reference *Chlorella variabilis* to map the proteins in the metabolic pathway based on their functional annotation. Mev software was used for the generation of heat-map.

### Multiple Reaction monitoring (MRM) assay

Based on the iTRAQ data analysis 6 proteins (Uniprot IDs: E1ZRS4, MDH; E1ZF27, PRK; E1Z6L2, SBP; E1ZKB3, TPI; E1ZRS1, RGP, and E1Z580, SOD) showing differential expression were validated using MRM based assay for relative quantitation. The respective FASTA sequences were fed into the Skyline v3.5 (MacCoss Lab Software- University of Washington) software to generate *in silico* trypsin digested peptides and respective MRM transitions with precursor ions of +2 and +3 charge and product ions of +1 and +2 charge. The transition lists were imported into LabSolutions software (Shimadzu Corporation, Japan) for screening. At least three unique peptides ranging in length from 8 to 20 amino acids and containing K/R tryptic ends with no miss-cleavages with 3–4 MRM transitions representing individual protein were considered. Unique peptides which were observed in iTRAQ data were prioritized during the peptide selection. For optimization of the runs, gel pieces representing the desired proteins were excised; in-gel digested, desalted and run on LCMS-8050 (Shimadzu Corporation, Japan). Once the parameters were optimized FC2 proteins (0 h, 40 h, 88 h and 120 h) spiked with 2 μg of BSA (internal standard) were run on the gel for 15 min and processed similarly. To account for possible changes in system response during the analysis, the external MS RT Calibration Mix (Cat# MSRT1-1VL, Sigma) was run. The results were imported into the Skyline v3.5 software, normalized with BSA and analyzed following the user manual.

### Western blot analysis

An aliquot of 40 μg of protein/well was separated on 12% SDS-PAGE and transferred onto PVDF membranes under semi-dry conditions using an ECL semi-dry transfer unit (GE Healthcare). Equal loading of the samples in each lane was confirmed by Ponceau staining of the transferred blots containing the resolved proteins. The western blot analysis was performed using 1:2000 dilution of anti-triosephosphate isomerase (Cat#Ab96696, abcam) and anti-rabbit-HRP conjugated antibody (Cat#62114038001A, GeNei) as secondary antibody (1:5000 dilution). The blot was developed as per the manufacturer’s protocol with the stable chromogen TMB-Western Blotting System (Cat# SB01, Invitrogen). Similarly, western blots were performed using anti-malate dehydrogenase, cytoplasmic (Cat# AS13 2706, Agrisera) and anti-phosphoribulokinase (Cat# AS07 257, Agrisera) primary antibodies, respectively with anti-rabbit-HRP-conjugated antibody as secondary antibody (1:5000 dilutions). The membranes were subjected to chromogenic detection using TMB/H_2_O_2_ reagent and scanned using Lab scan software. Densitometric analysis of the WB bands was performed with ImageQuant TL software (IQTL, GE Healthcare). The volume intensities obtained were expressed as mean ± standard deviation (n = 3).

## Additional Information

**How to cite this article:** Rai, V. *et al*. Real-time iTRAQ-based proteome profiling revealed the central metabolism involved in nitrogen starvation induced lipid accumulation in microalgae. *Sci. Rep.* **7**, 45732; doi: 10.1038/srep45732 (2017).

**Publisher's note:** Springer Nature remains neutral with regard to jurisdictional claims in published maps and institutional affiliations.

## Supplementary Material

Supplementary Information

## Figures and Tables

**Table 1:** Partial list of the differentially abundant proteins identified in FC2 exposed to N-starvation as a function of time.

Sl. No.	Acc_number	Entry_name	Gene ID	Uniq pep^a^	40/0 h	88/0 h	120/0 h
**Nitrogen assimilation, amino acid biosynthesis and Protein degradation**							
1.	E1Z7W6	Ferredoxin-dependent glutamate synthase, chloroplastic	CHLNCDRAFT_142154	3	1.167 ± 0.28	1.750 ± 0.97	1.716 ± 0.63
2.	E1Z4T9	Aspartate aminotransferase	CHLNCDRAFT_137913	1	1.443 ± 0.07	2.082 ± 0.23	2.289 ± 0.41
3.	A0A087SJX6	Argininosuccinate synthase	F751_3668	1	NS	2.314 ± 0.32	1.891 ± 0.26
4.	E1ZIW5	Arginine biosynthesis bifunctional protein ArgJ, chloroplastic	CHLNCDRAFT_135738		1.407 ± 0.55	NS	2.877 ± 2.32
5.	E1ZF33	Putative uncharacterized protein (arginine biosynthesis)	CHLNCDRAFT_52216	1	1.563 ± 0.96	1.797 ± 1.13	1.446 ± 0.64
6.	E1ZEF2	Cysteine synthase	CHLNCDRAFT_145435	3	1.317 ± 0.24	1.804 ± 0.03	2.065 ± 0.02
7.	E1ZP71	3-isopropylmalate dehydrogenase	CHLNCDRAFT_56307	1	1.190 ± 0.52	1.626 ± 0.75	1.485 ± 0.97
8.	E1Z6E6	Peptidyl-prolyl cis-trans isomerase	CHLNCDRAFT_29941	1	1.224 ± 0.47	2.051 ± 0.45	2.309 ± 0.71
9.	E1Z5I7	Ubiquitin-60S ribosomal protein L40-2	CHLNCDRAFT_48528	4	1.038 ± 0.39	1.930 ± 0.55	2.337 ± 0.70
10.	E1ZCR2	Proteasome subunit alpha type	CHLNCDRAFT_48764	1	0.987 ± 0.20	1.478 ± 0.38	1.741 ± 0.28
11.	A0A087SEY5	Proteasome subunit alpha type-4	F751_5131	1	1.035 ± 0.24	1.693 ± 0.13	2.566 ± 0.12
12.	E1Z575	Putative uncharacterized protein (Cytosol aminopeptidase)	CHLNCDRAFT_33905	1	1.493 ± 0.60	1.956 ± 0.50	2.047 ± 0.19
13.	E1ZMK0	Putative uncharacterized protein (metallo peptidase)	CHLNCDRAFT_137495	1	0.335 ± 0.07	0.835 ± 0.29	2.149 ± 1.37
14.	E1ZN67	Proteasome subunit alpha type	CHLNCDRAFT_26444	3	1.532 ± 0.02	1.666 ± 0.14	2.599 ± 0.33
15.	E1ZQQ9	Proteasome subunit alpha type	CHLNCDRAFT_27583	2	0.864 ± 0.49	1.676 ± 0.58	2.030 ± 0.85
16.	E1ZPZ3	Dihydroxy-acid dehydratase	CHLNCDRAFT_58991	2	1.419 ± 0.57	2.159 ± 1.25	2.117 ± 0.78
**Photosynthesis**							
17	E1Z6S6	Putative uncharacterized protein (photosystem II assembly)	CHLNCDRAFT_140330	1	1.451 ± 0.19	2.346 ± 0.38	2.684 ± 0.55
18	E1ZBP9	Ferredoxin–NADP reductase	CHLNCDRAFT_35035	3	1.312 ± 0.12	1.853 ± 0.19	2.121 ± 0.17
19	E1ZQR2	Putative uncharacterized protein	CHLNCDRAFT_32868	1	1.139 ± 0.11	2.508 ± 0.76	3.099 ± 0.70
20	E1ZPZ7	Putative uncharacterized protein	CHLNCDRAFT_139312	2	1.004 ± 0.17	1.781 ± 0.57	1.541 ± 0.11
21	E1ZRQ7	Porphobilinogen deaminase, chloroplastic	CHLNCDRAFT_33052	2	0.775 ± 0.23	1.153 ± 0.30	0.962 ± 0.14
**Carbon metabolism**							
22	F2YGL1	Large subunit of Rubisco	rbcL	3	1.181 ± 0.40	1.422 ± 0.61	1.589 ± 0.48
23	A0A087SAW7	Ribulose bisphosphate carboxylase small chain	F751_5580	1	1.439 ± 0.19	1.961 ± 0.33	2.192 ± 0.12
24	E1ZRS4	Malate dehydrogenase^(b),(c),(d)^	CHLNCDRAFT_59812	3	1.539 ± 0.28	2.308 ± 0.47	3.312 ± 0.17
25	E1ZT20	Glyceraldehyde-3-phosphate dehydrogenase	CHLNCDRAFT_49269	2	1.073 ± 0.31	1.604 ± 0.24	1.159 ± 0.25
26	E1ZQQ5	Fructose-bisphosphate aldolase	CHLNCDRAFT_37179	2	1.643 ± 0.86	1.647 ± 0.73	3.292 ± 2.08
27	E1ZF27	Phosphoribulokinase^(c),(d)^	CHLNCDRAFT_31168	3	1.280 ± 0.05	1.363 ± 0.22	1.137 ± 0.17
28	A0A087SU66	Pyruvate kinase	F751_1646		1.418 ± 0.18	2.289 ± 0.01	1.980 ± 0.14
29	E1Z2U6	Phosphoenolpyruvate carboxykinase [ATP] 1	CHLNCDRAFT_56532	2	1.147 ± 0.66	2.179 ± 1.05	2.947 ± 0.71
30	E1Z5A0	Phosphoglycerate kinase	CHLNCDRAFT_29609	2	1.596 ± 0.25	2.381 ± 0.65	3.144 ± 0.71
31	E1Z6L2	Sedoheptulose-1,7-bisphosphatase^(b),(d)^	CHLNCDRAFT_19601	2	0.437 ± 0.19	0.638 ± 0.15	0.723 ± 0.09
32	E1Z7C4	Putative uncharacterized protein (Ribose-5-phosphate isomerase)^(b),(d)^	CHLNCDRAFT_34303	1	1.164 ± 0.11	2.164 ± 0.77	2.584 ± 0.15
33	E1Z7S4	Ribulose-phosphate 3-epimerase	CHLNCDRAFT_56033	1	0.800 ± 0.02	1.015 ± 0.16	1.642 ± 0.33
34	E1ZKS0	Enolase	CHLNCDRAFT_136652	2	1.688 ± 0.12	2.244 ± 0.81	1.723 ± 1.50
35	E1Z1Z7	Putative uncharacterized protein	CHLNCDRAFT_29144		1.022 ± 0.44	1.433 ± 0.03	3.032 ± 0.67
36	E1ZKB3	Triosephosphate isomerase^(b),(c),(d)^	CHLNCDRAFT_36334	2	1.223 ± 0.69	2.184 ± 1.52	2.232 ± 0.96
37	E1ZRS1	Reversibly glycosylated protein^(b),(d)^	CHLNCDRAFT_56392	3	1.736 ± 0.11	3.077 ± 0.15	3.329 ± 0.20
**Fatty acid metabolism**							
38	E1Z5W8	Acyl carrier protein	CHLNCDRAFT_29840	1	0.757 ± 0.28	1.499 ± 0.75	1.618 ± 0.42
39	E1Z2Y2	Putative uncharacterized protein (enoyl-[acyl-carrier protein] reductase I)	CHLNCDRAFT_59537	1	NS	NS	2.319 ± 1.61
40	E1Z8J0	Putative uncharacterized protein (hydro-lyase activity)	CHLNCDRAFT_34566	1	0.964 ± 0.09	1.472 ± 0.27	2.131 ± 0.32
41	E1ZIL0	Putative uncharacterized protein (Acyl-CoA dehydrogenase)	CHLNCDRAFT_24792	1	2.195 ± 0.49	1.779 ± 0.02	2.151 ± 0.09
42	E1ZES7	Putative uncharacterized protein (phosphotransferase)	CHLNCDRAFT_57872	1	1.382 ± 0.63	2.135 ± 2.29	2.539 ± 2.60
**Stress responsive**							
43	E1Z580	Superoxide dismutase^(d)^	CHLNCDRAFT_33910	4	1.000 ± 0.57	1.928 ± 0.89	2.152 ± 1.18
44	E1ZG17	Putative uncharacterized protein (Fragment) (peroxidin activity)	CHLNCDRAFT_23497	2	1.327 ± 0.36	2.203 ± 0.82	2.108 ± 0.73
45	E1ZCK9	Putative uncharacterized protein (Chloroplastic lipocalin)	CHLNCDRAFT_145104	2	1.398 ± 0.60	1.773 ± 0.41	2.209 ± 0.30
46	A0A087SDD7	Octanoyltransferase	F751_0955	1	1.379 ± 0.44	1.963 ± 0.87	2.809 ± 0.97
47	A0A087SKJ2	Serine hydroxymethyltransferase	F751_4739	1	1.551 ± 0.53	1.871 ± 1.08	2.940 ± 2.44
48	E1Z357	Serine-glyoxylate aminotransferase	CHLNCDRAFT_33614	1	1.161 ± 0.55	1.958 ± 0.26	1.782 ± 0.41

^(a)^Mean value for the identified unique peptides in different replicates is represented. ^(b)^Differential abundance for these candidates is also identified in DIGE. ^(c)^These candidates are validated by Western blotting. ^(d)^These candidates are validated by MRM. NS: Not significant.

**Table 2:** Modulation of various physiological pathways in *Chlorella* sp. FC2 IITG starved with nitrogen.

Sl. No.	Pathways	Observed no. of candidates	Gene IDs of test set in subcategory	Putative role in nitrogen-starvation induced lipid accumulation in microalgae	References
1	Protein degradation	8	E1ZN67, E1ZQQ9, E1Z575, E1ZMK0, E1Z5I7, E1ZCR2, A0A087SEY5, E1ZR38	Enhanced protein degradation is required to maintain the intracellular nitrogen homeostasis.	6,49,50
2	Amino acid biosynthesis	9	E1ZPZ3, E1Z4T9, E1ZF33, A0A087SJX6, A0A087SKJ2, E1ZEF2, E1ZIW5, E1Z357, E1ZP71	To maintain the overall intracellular levels of nitrogen, amino acid biosynthesis particularly of aspartate, glutamate and arginine is elevated. Accumulation of arginine is the strategy of the microalgal cells to trap maximal nitrogen (3 N-atoms in this case).	11,47
3	Photosynthesis	9	E1ZRQ7, A0A087SAW7, E1Z6S6, E1ZBP9, E1ZFB3, A0A087SP02, E1ZQR2, F2YGR0, E1ZPZ7	Nitrogen starvation often leads to chlorosis. Likewise, in the present study elevated levels of porphobilinogen deaminase degrades chlorophyll, which is mirrored in the physiological data. In fact chlorophyll degradation allows recycling of nitrogen and other nutrients and protects cell from building-up of phototoxic chlorophyll intermediates. Interestingly most of the other proteins associated with light-reaction were up-regulated, suggesting enhanced NADPH and ATP synthesis needed for sustaining stress, however owing to chlorophyll degradation the net yield is lowered.	51,55,56,98, 99, 100, 101
4	Carbohydrate metabolism	20	E1Z5R5, E1ZCC9, E1ZT20, A0A087SEW0, A0A087SHU5, A0A087SU66, E1Z2U6, E1Z5A0, E1Z6L2, E1Z7C4, E1Z7S4, E1ZF27, E1ZKS0, E1ZN90, E1ZQQ5, E1ZRS4, F2YGL1, E1Z1Z7, E1ZKB3, E1ZRS1	Several of the enzymes associated with glycolysis, reductive PPP and TCA cycle are up-regulated to fulfil the energy needs of the N-starved FC2 cells. The glycolytic enzymes viz. GPDH, PGK, ENL and PK has role in the generation of ATP, reducing equivalents and pyruvate (convertible to fatty acid). Besides the TCA intermediates may be transaminated to different amino acids.	11,58,59,64,68,102
5	Fatty acid metabolism	5	E1Z5W8, E1Z2Y2, E1Z8J0, E1ZES7, E1ZIL0	Several studies have suggested the up-regulation of the transpricts and metabolites associated with the fatty acid biosynthesis during nitrogen starvation. Additionally, recycling of the fatty acids from the existing membrane lipids via β-oxidation leads to overall increase in intracellular fatty acid repertoire. The fatty acid reserves are prime contributors in TAG biosynthesis.	68,103,104
6	Calcium hemeostsis	4	E1ZTD2, A0A087S9V6, A0A087SLE3, E1ZSB3	Imbalance of Ca homeostasis leads to ER stress leading to the activation of the unfolded protein response (UPR). Activated UPR either reduce protein translation or increase ER-associated protein degradation. Enhanced protein degradation is required for sustained levels of intracellular nitrogen.	105
7	Stress responsive	6	E1Z580, E1ZG17, E1ZCK9, A0A087SDD7, A0A087SKJ2, E1Z357	Abiotic stress induces oxidative damage in algae; hence stress responsive genes are up-regulated to countact the effect. Photorespiration being a part and partial of oxidative stress scavenging system in green tissues is also activated in response to nitrogen starvation.	106,107
