# Supplementary material for: Real-time iTRAQ-based proteome profiling revealed the central metabolism involved in nitrogen starvation induced lipid accumulation in microalgae
Source: Sci Rep. 2017 Apr 5;7:45732. doi: 10.1038/srep45732 (PMC5381106; doi:10.1038/srep45732)
Supplement: Supplementary Information [file srep45732-s1.pdf]

Supplementary Figures

Fig. S1: DIGE gel images **a.** N+ Vs N-40 **b.** N+ Vs N-88 **c.** N+ Vs N-120

Fig. S2: Differentially expressed protein spots in DIGE analysis (marked by arrow). The image displayed is the master gel showing the highest number of spots.

Fig. S3: Functional categorization of differentially expressed proteins of *Chlorella* sp. FC2 IITG during different N-starvation time points (0 h, 40 h, 88 h and 120 h)

Fig. S4: Pipeline for Multiple Reaction Monitoring (MRM) analysis, and the MRM traces of 25 peptides representing 6 different proteins and BSA (internal quality control) each represented by 3 or more unique peptides. %RSD for BSA was less than 20.

Fig. S5: Schematic representation of the enzymes namely GPDH, PGK, ENL, FBP, ALDO, TPI, and PK belonging to glycolytic pathway, and RPI and RPE from non-oxidative PPP functioning cordially to generate NADPH and pyruvate required for neutral lipid biosynthesis.

Fig. S1 A: DIGE gel images of Group I (N+ Vs N-40)

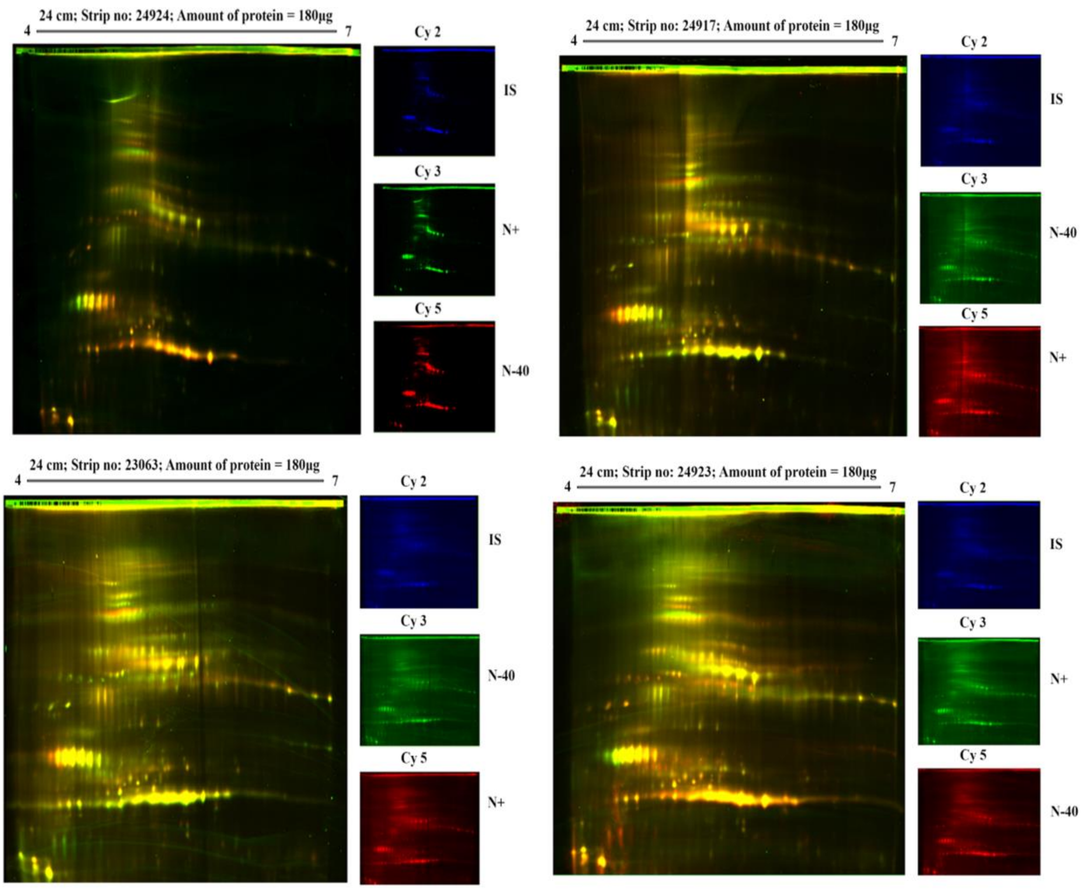

Fig. S1 B: DIGE gel images of Group II (N+ Vs N-88)

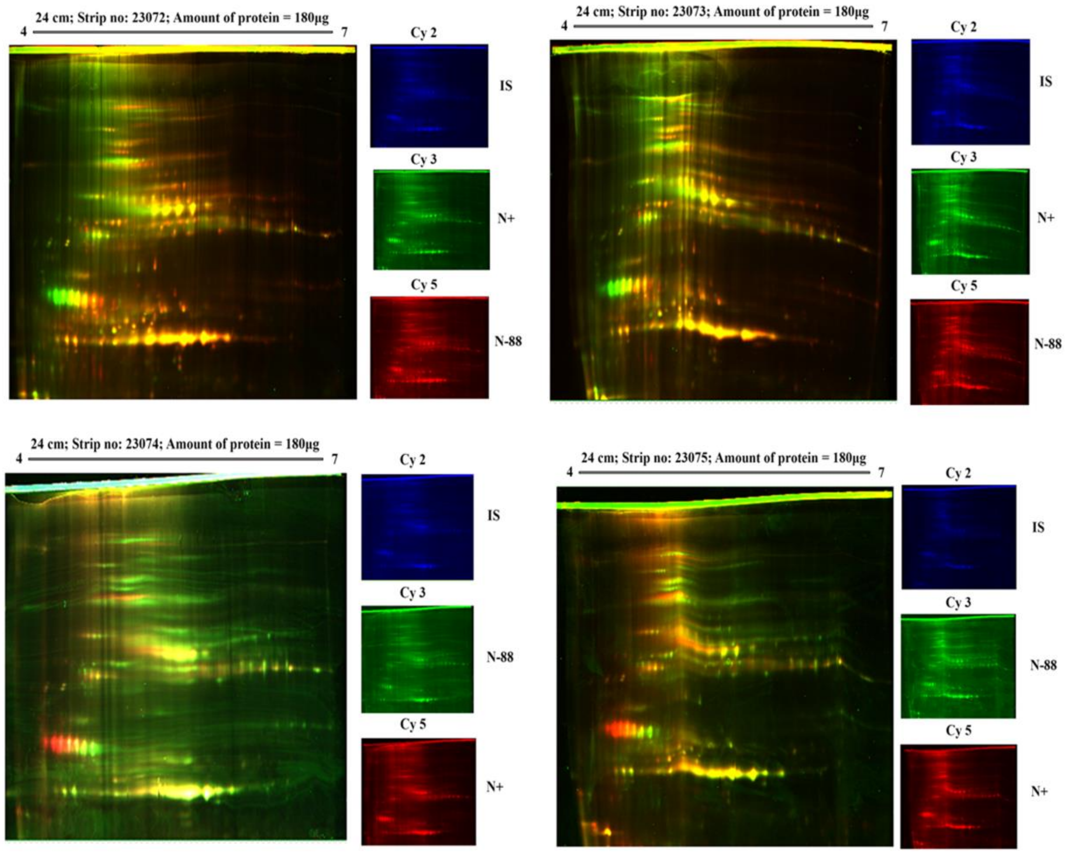

Fig. S1 C: DIGE gel images of Group III (N+ Vs N-120)

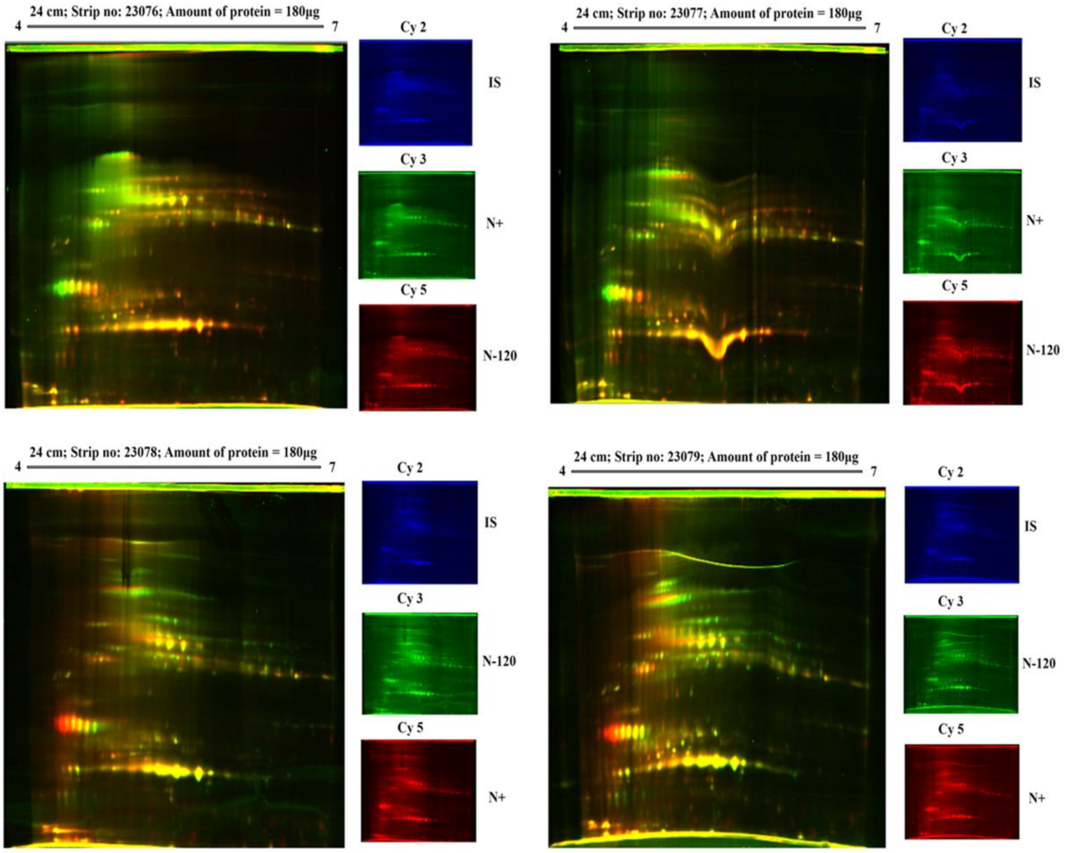

Fig. S2: Differentially expressed protein spots in DIGE analysis (marked by arrow). The image displayed is the master gel showing the highest number of spots.

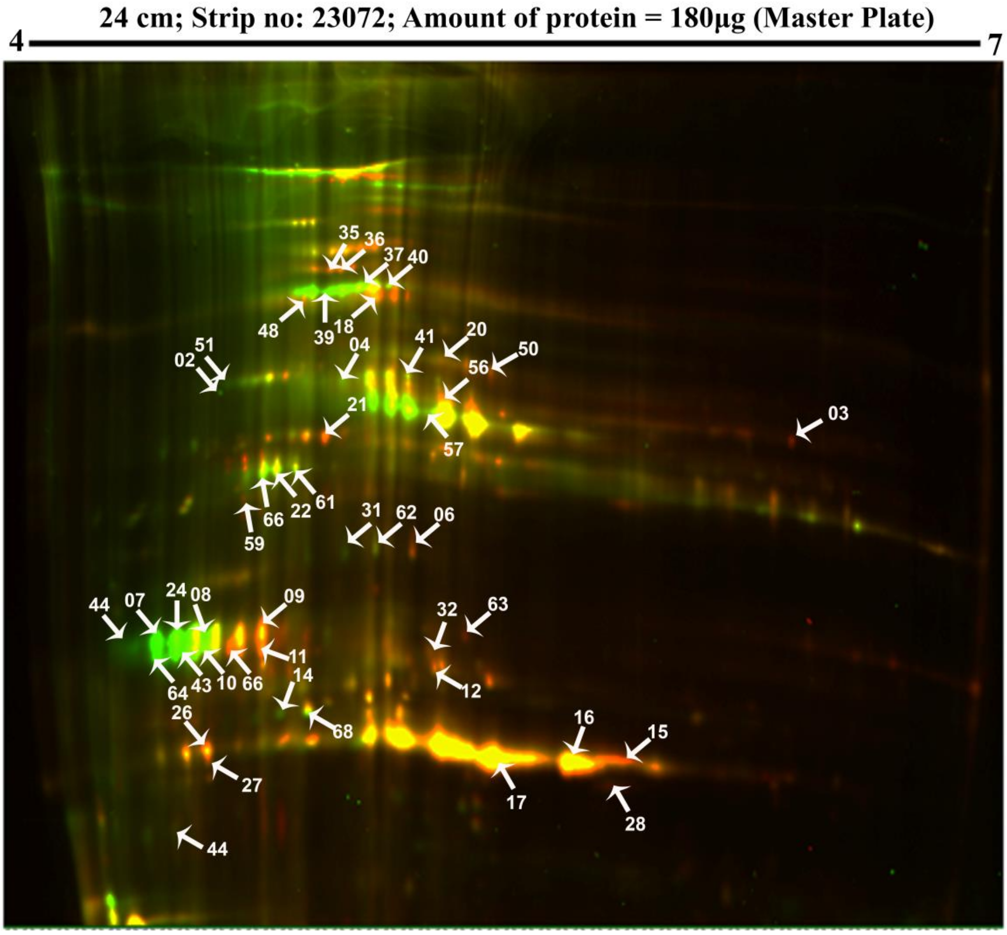

Fig. S3: Functional categorization of differentially expressed proteins of *Chlorella* sp. FC2 IITG during different N-starvation time points (0 h, 40 h, 88 h and 120 h)

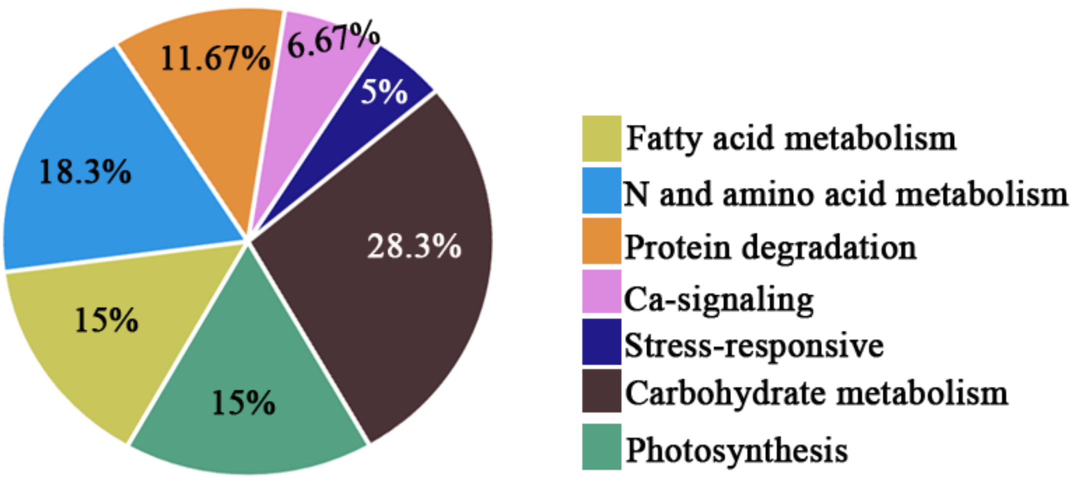

Fig. S4: Pipeline for Multiple Reaction Monitoring (MRM) analysis, and the MRM traces of 25 peptides representing 6 different proteins and BSA (internal quality control) each represented by 3 or more unique peptides. %RSD for BSA was less than 20.

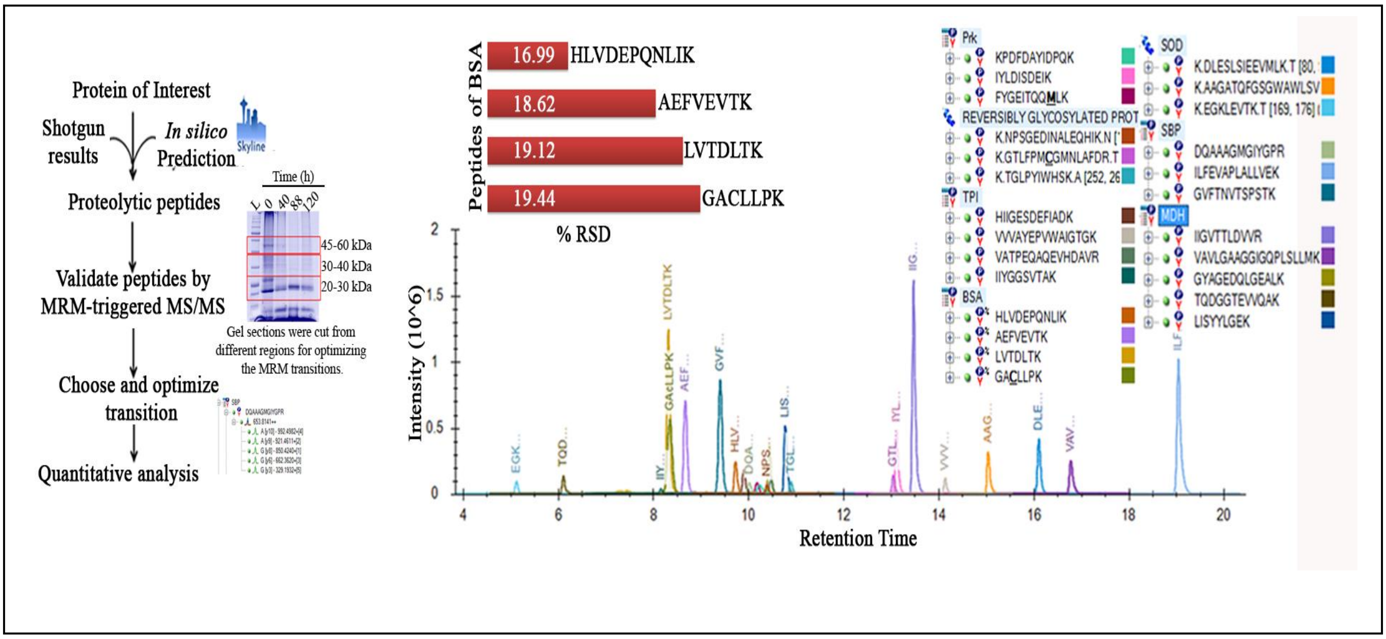

Fig. S5: Schematic representation of the enzymes namely GPDH, PGK, ENL, FBP, ALDO, TPI, and PK belonging to glycolytic pathway, and RPI and ribulose-phosphate-3-epimerase (RPE) from non-oxidative PPP functioning cordially to generate NADPH and pyruvate required for neutral lipid biosynthesis.

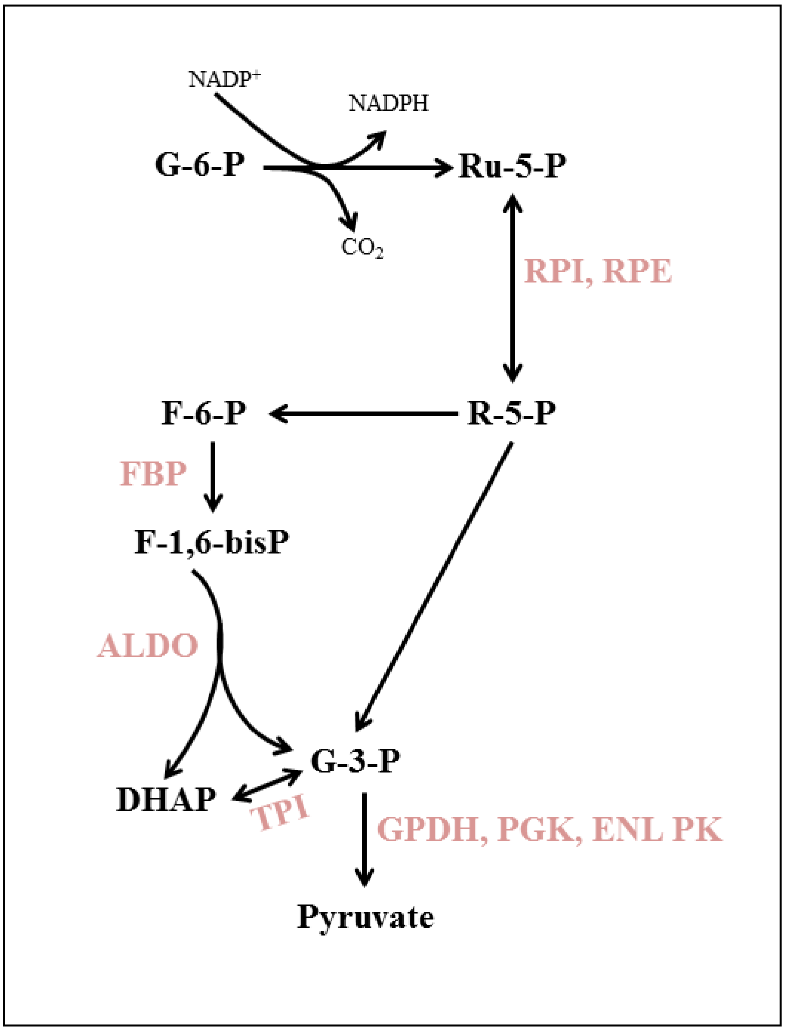

Supplementary Tables

Table S1: DIGE experimental design, N+: N-sufficient condition; N-40: N-starvation for 40 h; N-88: N-starvation for 88 h and N-120: N-starvation for 120 h

Table S2: A. Spots up-regulated in test (N-40, N-88 and N-120) as compared to control (N+) in DIGE analysis. B. Spots down-regulated in test (N-40, N-88 and N-120) as compared to control (N+) in DIGE analysis

Table S3: Complete list of Q-TOF identified differentially abundant proteins in N-starved *Chlorella* sp. FC2 IITG detected in DIGE analysis. Rows highlighted in yellow represents the protein spots unique DIGE, while others are common to iTRAQ.

Table S4: Complete list of the differentially abundant proteins identified in FC2 exposed to N-starvation as a function of time using iTRAQ-based quantitative proteomics and Q-TOF mass spectrometer.

Table S5: The score, pI, molecular ion, Q1/Q3 transitions, and optimized CE for each peptide obtained in MRM study

Table S1: DIGE experimental design, N+: N-sufficient condition; N-40: N-starvation for 40 h; N-88: N-starvation for 88 h and N-120: N-starvation for 120 h

	Sl. No.	Cy 2 (Internal Standard)	Cy 3	Cy 5
Group I	1	$N^+120+N^-120+N^-88+N^-40$	N^+	N^-40
	2	$N^+120+N^-120+N^-88+N^-40$	N^+	N^-40
	3	$N^+120+N^-120+N^-88+N^-40$	N^-40	N^+
	4	$N^+120+N^-120+N^-88+N^-40$	N^-40	N^+

	Sl. No.	Cy 2 (Internal Standard)	Cy 3	Cy 5
Group II	1	$N^+120+N^-120+N^-88+N^-40$	N^+	N^-88
	2	$N^+120+N^-120+N^-88+N^-40$	N^+	N^-88
	3	$N^+120+N^-120+N^-88+N^-40$	N^-88	N^+
	4	$N^+120+N^-120+N^-88+N^-40$	N^-88	N^+

	Sl. No.	Cy 2 (Internal Standard)	Cy 3	Cy 5
Group III	1	$N^+120+N^-120+N^-88+N^-40$	N^+	N^-120
	2	$N^+120+N^-120+N^-88+N^-40$	N^+	N^-120
	3	$N^+120+N^-120+N^-88+N^-40$	N^-120	N^+
	4	$N^+120+N^-120+N^-88+N^-40$	N^-120	N^+

Table S2 A: Spots up-regulated in test (N-40, N-88 and N-120) as compared to control (N+) in DIGE analysis

2D-DIGE up-regulated spots						
**Sl.No.**	**Position**	**Master Plate**	**Appearance**	**T-test**	**1-ANOVA**	**Av. Ratio**
1	14	523	30 (30)	1.60E-05	3.60E-07	3.07
2	65	468	30 (30)	2.50E-05	2.30E-07	2.71
3	2	296	21 (30)	7.30E-05	1.90E-04	2.55
4	62	443	18 (30)	0.0045	0.0006	2.42
5	45	164	24 (30)	6.40E-04	2.50E-03	2.35
6	27	557	30 (30)	1.40E-04	7.00E-07	2.29
7	1	282	21 (30)	5.10E-03	2.40E-02	2.18
8	48	257	21 (30)	2.60E-03	4.50E-03	2.08
9	8	458	27 (30)	0.00037	1.60E-03	2.05
10	49	262	18 (30)	0.00018	0.00028	1.97
11	34	496	24 (30)	0.00018	3.30E-04	1.91
12	46	229	24 (30)	4.60E-05	2.60E-04	1.91
13	9	464	27 (30)	0.00018	2.80E-04	1.89
14	41	310	27 (30)	4.60E-05	1.30E-07	1.86
15	11	478	24 (30)	6.40E-04	1.60E-03	1.73
16	66	479	21 (30)	8.60E-05	0.00019	1.69
17	32	481	24 (30)	0.0005	3.50E-04	1.68
18	18	252	27 (30)	1.30E-04	1.10E-06	1.67
19	37	248	30 (30)	4.60E-05	7.60E-07	1.63
20	42		30 (30)	0.00067	1.50E-03	1.62
21	38	249	30 (30)	4.60E-05	2.10E-08	1.6
22	40	251	27 (30)	1.70E-04	7.80E-06	1.6
23	43	472	30 (30)	0.064	5.90E-04	1.6
24	67	493	24 (30)	0.0021	0.013	1.56
25	31	444	24 (30)	0.00043	5.70E-06	1.54
26	10	467	30 (30)	0.0016	1.10E-02	1.52
27	53	314	18 (30)	0.0051	1.30E-02	1.51
28	33	483	30 (30)	0.0051	2.10E-08	1.43
29	39	250	30 (30)	8.90E-05	8.80E-06	1.41
30	21	372	30 (30)	0.031	1.20E-06	1.4
31	35	228	30 (30)	4.00E-03	1.10E-07	1.4

Table S2 B: Spots down-regulated in test (N-40, N-88 and N-120) as compared to control (N+) in DIGE analysis

2D-DIGE down-regulated spots						
**Sl.No.**	**Position**	**Master Plate**	**Appearance**	**T-test**	**1-ANOVA**	**Av. Ratio**
1	25	469	30 (30)	1.60E-05	7.90E-10	4.39
2	63	459	30 (30)	1.60E-05	1.30E-10	4.16
3	23	421	21 (30)	0.00017	7.40E-06	3.69
4	30	348	15 (30)	3.90E-03	1.60E-05	3.25
5	24	456	27 (30)	0.0002	1.20E-08	2.6
6	28	558	27 (30)	0.00051	1.80E-08	2.57
7	5	349	21 (30)	2.00E-04	5.90E-04	2.24
8	57	345	27 (30)	4.60E-05	1.20E-05	2.13
9	3	312	21 (30)	1.80E-04	2.00E-03	2.08
10	7	457	24 (30)	5.40E-05	5.10E-06	2.07
11	29	287	27 (30)	7.80E-04	1.80E-08	2.05
12	4	316	21 (30)	0.00068	6.60E-04	2.04
13	60	417	24 (30)	0.00017	7.80E-06	1.98
14	16	531	24 (30)	4.60E-05	2.30E-05	1.84
15	6	442	21 (30)	0.00018	6.00E-04	1.8
16	15	526	30 (30)	9.70E-05	1.10E-05	1.77
17	17	541	30 (30)	7.30E-05	2.40E-06	1.77
18	20	281	27 (30)	1.80E-04	0.00046	1.72
19	22	416	24 (30)	7.20E-04	5.10E-06	1.71
20	26	545	30 (30)	0.0018	4.60E-07	1.71
21	44	455	21 (30)	0.0033	0.015	1.7
22	36	230	30 (30)	1.80E-04	7.00E-07	1.67
23	13	503	21 (30)	0.00032	1.50E-04	1.65
24	52	302	24 (30)	0.00098	2.20E-04	1.64
25	54	318	24 (30)	0.0019	9.00E-04	1.6
26	59	415	24 (30)	0.00031	0.00082	1.59
27	64	466	27 (30)	0.0075	1.30E-10	1.56
28	58	351	21 (30)	0.0026	0.0023	1.54
29	55	335	24 (30)	9.70E-05	0.00015	1.53
30	19	266	27 (30)	2.50E-05	1.30E-05	1.5
31	12	488	30 (30)	0.0043	4.10E-06	1.49
32	56	343	27 (30)	0.00017	0.00048	1.48
33	61	423	27 (30)	0.0017	3.30E-06	1.48
34	47	234	27 (30)	5.70E-02	2.00E-01	1.45
35	68	519	30 (30)	0.0029	0.00013	1.26
36	51	300	24 (30)	0.0035	2.60E-02	1.22
37	50	277	30 (30)	0.061	0.0017	1.2

Table S3: Complete list of Q-TOF identified differentially abundant proteins in N-starved *Chlorella* sp. FC2 IITG detected in DIGE analysis. Rows highlighted in yellow represents the protein spots unique DIGE, while others are common to iTRAQ.

Sl. No.	Spot ID	Master Plate ID	Protein Accession No and Name with NCBI_nr	Species	Protein Score	MW (kDa)	No. of peptides	Peptide Sequence
1	10	467	(gi|159467673) ribose-5-phosphate isomerase	*Chlamydomonas reinhardtii*	134	29.029	7	YTLQR; AVEYVK; LVEGLGGSK; FVCIVDDSK; LAMPVEIVQFCHK; SGMVVGLGTGSTAAFAVDR; LDVAIDGADEVDPNLDVVK
2	12	488	(gi|81157955) triosephosphate isomerase cytoplasmic type	*Chlorella pyrenoidosa*	259	26.932	12	VAYALSK; IQDWSK; SNEELVK; IIYGGSVTAK; EAGNTLDVNAR; GLGVIYCIGEK; ALADKIQDWSK; VVVAYEPVWAIGTGK; VATPEQAQEVHDAVR; LEEREAGNTLDVNAR; VATPEQAQEVHDAVRK; NCGDLATQPDIDGFLVGGASLKPEFVDIIK
3	22	416	(gi|384244700) sedoheptulose-1,7-bisphosphatase	*Coccomyxa subellipsoidea* C-169	185	37.056	6	LLFEALK; MFSPGNLR; GVFTNVTSPSTK; DQAAAGMGIYGPR; EKGVFTNVTSPSTK; YTGGMVPDVFQIIVK
4	27	557	(gi|384246944) thioredoxin-like protein	*Coccomyxa subellipsoidea* C-169	74	29.204	4	GLFLIDK; SVDETLR; EGVIQHATINNLAFGR; GLFLIDKEGVIQHATINNLAFGR
5	36	230	(gi|134035494) alpha-tubulin	*Gossypium hirsutum*	230	50.304	6	LSVDYGKK; TVQFVDWCPTGFK; AVFVDLEPTVIDEVR; AFVHWYVGEGMEEGEFSEAR; FDGAINVDITEFQTNLVPYPR; RAFVHWYVGEGMEEGEFSEAR

6	37	248	(gi|307103200) reversibly glycosylated protein	*Chlorella variabilis*	228	39.524	10	GYPFSMR; AWCISFK; YVDAVMTIPK; TGLPYIWHSK; YDDMWAGWCVK; DVFSCYLELAEK; YVYTIDDDCFVAK; NPSGEDINALEQHIK; GTLFPMCGMNLAFDR; APPLADQLDIVIPTIR
7	43	472	(gi|288816141) oxygen-evolving enhancer protein	Bornetella sphaerica	86	15.546	5	GSTFLDPK; VPFLFTVK; FCMEPTSFTVK; LTYTLDDMSGTFK; FCMEPTSFTVKEESQFK
8	56	343	(gi|159485250) fructose-1,6-bisphosphate aldolase	*Chlamydomonas reinhardtii*	227	41.301	7	ALQNTVLK; DCAYGLAR; GILAMDESNATCGK; LDSIGVENTEENR; LDSIGVENTEENRR; GILAMDESNATCGKR; YAAIAQNAGLVPIVEPEVLLDGEHDIDR
9	64	466	(gi|384247377) putative 40S ribosomal protein S3, partial	*Coccomyxa subellipsoidea* C-169	62	24.272	4	QGVLGIK; ACYGVLR; TQNVLGVK; GCEVIVSGK
10	3	312	(gi|159490405) malate dehydrogenase	*Chlamydomonas reinhardtii*	179	38.827	5	DDLFK; TFYAEK; AKTFYAEK; TQDGGTEVVQAK; RTQDGGTEVVQAK
11	44	572	(gi|161728799) 33kDa oxygen evolving protein of photosystem II	*Hafniomonas montana*	49	30.872	1	MCMEPTSFTVK
12	48	257	(gi|295917894) beta-cyanoalanine synthase/ Cysteine synthase	*Triticum aestivum*	112	40.22	1	AFGAQLVLTDPAK
13	51	300	(gi|20331) actin	*Oryza sativa Indica Group*	41	42.024	1	TTGMVLDSGDGVTHTVPIYEGYALPHAILR

Table S4: Complete list of the differentially abundant proteins identified in FC2 exposed to N-starvation as a function of time using iTRAQ-based quantitative proteomics and Q-TOF mass spectrometer.

Acc_number	Entry_name	Gene ID	40/0 hr	88/0 hr	120/0 hr
E1ZE73	UDP-GlcNAc:betaGal beta-1,3-N-acetylglucosaminyltransferase 6	CHLNCDRAFT_145336	1.062 ± 0.27	1.680 ± 0.59	1.723 ± 0.41
E1Z6E6	Peptidyl-prolyl cis-trans isomerase	CHLNCDRAFT_29941	1.224 ± 0.47	2.051 ± 0.45	2.309 ± 0.71
E1ZPZ3	Dihydroxy-acid dehydratase	CHLNCDRAFT_58991	1.419 ± 0.57	2.159 ± 1.25	2.117 ± 0.78
E1Z4T9	Aspartate aminotransferase	CHLNCDRAFT_137913	1.443 ± 0.07	2.082 ± 0.23	2.289 ± 0.41
E1ZF33	Putative uncharacterized protein (arginine biosynthesis)	CHLNCDRAFT_52216	1.563 ± 0.96	1.797 ± 1.13	1.446 ± 0.64
A0A087SJX6	Argininosuccinate synthase	F751_3668	NS	2.314 ± 0.32	1.891 ± 0.26
A0A087SKJ2	Serine hydroxymethyltransferase	F751_4739	1.551 ± 0.53	1.871 ± 1.08	2.940 ± 2.44
E1ZEF2	Cysteine synthase	CHLNCDRAFT_145435	1.317 ± 0.24	1.804 ± 0.03	2.065 ± 0.02
E1ZIW5	Arginine biosynthesis bifunctional protein ArgJ, chloroplastic	CHLNCDRAFT_135738	1.407 ± 0.55	NS	2.877 ± 2.32
E1Z357	Serine-glyoxylate aminotransferase	CHLNCDRAFT_33614	1.161 ± 0.55	1.958 ± 0.26	1.782 ± 0.41
E1ZP71	3-isopropylmalate dehydrogenase	CHLNCDRAFT_56307	1.190 ± 0.52	1.626 ± 0.75	1.485 ± 0.97
E1ZTD2	Putative uncharacterized protein (Calcium-transporting ATPase)	CHLNCDRAFT_59377	1.301	2.661 ± 0.34	2.990 ± 0.67
A0A087S9V6	Calmodulin	F751_0028	0.885 ± 0.50	NS	2.382 ± 0.60
A0A087SLE3	Calcium-transporting ATPase	F751_2134	1.502 ± 0.25	2.571 ± 1.05	2.784 ± 0.99
E1ZSB3	Putative uncharacterized protein (Calmodulin)	CHLNCDRAFT_59820	1.023 ± 0.10	1.621 ± 0.07	1.940 ± 0.06
E1Z5R5	Putative uncharacterized protein (malate dehydrogenase)	CHLNCDRAFT_140591	1.146 ± 0.10	1.765 ± 0.69	2.154 ± 0.34

E1ZCC9	Putative uncharacterized protein (hydrolyses carbon-nitrogen bond)	CHLNCDRAFT_57507	1.274 ± 0.12	3.202 ± 0.64	2.723 ± 0.64
E1ZT20	Glyceraldehyde-3-phosphate dehydrogenase	CHLNCDRAFT_49269	1.073 ± 0.31	1.604 ± 0.24	1.159 ± 0.25
A0A087SEW0	Alpha-1,4 glucan phosphorylase	F751_5106	1.120 ± 0.26	1.469 ± 0.05	1.799 ± 0.09
A0A087SHU5	Fructose-1,6-bisphosphatase, chloroplastic	F751_3979	0.923 ± 0.18	1.003 ± 0.26	1.445 ± 0.09
A0A087SU66	Pyruvate kinase	F751_1646	1.418 ± 0.18	2.289 ± 0.01	1.980 ± 0.14
E1Z2U6	Phosphoenolpyruvate carboxykinase [ATP] 1	CHLNCDRAFT_56532	1.147 ± 0.66	2.179 ± 1.05	2.947 ± 0.71
E1Z5A0	Phosphoglycerate kinase	CHLNCDRAFT_29609	1.596 ± 0.25	2.381 ± 0.65	3.144 ± 0.71
E1Z6L2	Sedoheptulose-1,7-bisphosphatase, chloroplastic	CHLNCDRAFT_19601	0.437 ± 0.19	0.638 ± 0.15	0.723 ± 0.09
E1Z7C4	Putative uncharacterized protein (Ribose-5-phosphate isomerase)	CHLNCDRAFT_34303	1.164 ± 0.11	2.164 ± 0.77	2.584 ± 0.15
E1Z7S4	Ribulose-phosphate 3-epimerase	CHLNCDRAFT_56033	0.800 ± 0.02	1.015 ± 0.16	1.642 ± 0.33
E1ZF27	Phosphoribulokinase	CHLNCDRAFT_31168	1.280 ± 0.05	1.363 ± 0.22	1.137 ± 0.17
E1ZKS0	Enolase	CHLNCDRAFT_136652	1.688 ± 0.12	2.244 ± 0.81	1.723 ± 1.50
E1ZN90	Malate dehydrogenase	CHLNCDRAFT_32420	1.744 ± 0.32	NS	1.947 ± 1.69
E1ZQQ5	Fructose-bisphosphate aldolase	CHLNCDRAFT_37179	1.643 ± 0.86	1.647 ± 0.73	3.292 ± 2.08
E1ZRS4	Malate dehydrogenase	CHLNCDRAFT_59812	1.539 ± 0.28	2.308 ± 0.47	3.312 ± 0.17
F2YGL1	Large subunit of Rubisco	rbcL	1.181 ± 0.40	1.422 ± 0.61	1.589 ± 0.48
E1Z1Z7	Putative uncharacterized protein	CHLNCDRAFT_29144	1.022 ± 0.44	1.433 ± 0.03	3.032 ± 0.67
E1ZKB3	Triosephosphate isomerase cytoplasmic type	CHLNCDRAFT_36334	1.223 ± 0.69	2.184 ± 1.52	2.232 ± 0.96
E1ZRS1	Reversibly glycosylated protein	CHLNCDRAFT_56392	1.736 ± 0.11	3.077 ± 0.15	3.329 ± 0.20
E1ZSZ8	Putative uncharacterized protein	CHLNCDRAFT_141499	0.808 ± 0.18	1.742 ± 0.47	1.412 ± 0.17
E1Z4B1	Cytosine-specific methyltransferase	CHLNCDRAFT_137705	0.694 ± 0.07	0.594 ± 0.19	0.727 ± 0.27
A0A087SQ61	E1A-binding protein p400	F751_5430	1.904 ± 0.20	2.568 ± 0.27	1.594 ± 0.41
E1ZFV0	Histone H4	CHLNCDRAFT_134363	1.620 ± 0.17	2.878 ± 0.59	1.069 ± 0.36
E1ZH25	Putative uncharacterized protein	CHLNCDRAFT_134898	1.470 ± 0.46	1.653 ± 0.16	2.551 ± 0.04
E1ZIK7	Putative uncharacterized protein	CHLNCDRAFT_135612	0.965 ± 0.24	1.740 ± 0.32	2.422 ± 0.11

E1ZMP7	Putative uncharacterized protein (Fragment) (ATP binding)	CHLNCDRAFT_13557	NS	1.759 ± 0.43	2.462 ± 0.83
E1ZJJ7	Putative uncharacterized protein	CHLNCDRAFT_58355	NS	2.608 ± 1.09	2.982 ± 0.67
A0A087SJF6	Trafficking protein particle complex subunit 2-like protein	F751_1474	0.558 ± 0.10	1.842 ± 0.47	2.240 ± 0.50
A0A087SBZ3	Cytochrome c	F751_3438	1.812 ± 0.28	4.025 ± 1.24	2.370 ± 0.70
A0A087SDD7	Octanoyltransferase	F751_0955	1.379 ± 0.44	1.963 ± 0.87	2.809 ± 0.97
E1Z5W8	Acyl carrier protein	CHLNCDRAFT_29840	0.757 ± 0.28	1.499 ± 0.75	1.618 ± 0.42
E1Z8J0	Putative uncharacterized protein	CHLNCDRAFT_34566	0.964 ± 0.09	1.472 ± 0.27	2.131 ± 0.32
A0A087SK58	Erythronolide synthase, modules 3 and 4	F751_6263	1.404 ± 0.59	2.511 ± 0.49	3.776 ± 0.04
E1ZIL0	Putative uncharacterized protein (Acyl-CoA dehydrogenase)	CHLNCDRAFT_24792	2.195 ± 0.49	1.779 ± 0.02	2.151 ± 0.09
E1Z2Y2	Putative uncharacterized protein (enoyl-[acyl-carrier protein] reductase I)	CHLNCDRAFT_59537	NS	NS	2.319 ± 1.61
E1ZGF5	Putative uncharacterized protein	CHLNCDRAFT_134507	0.983 ± 0.08	1.164 ± 0.16	1.774 ± 0.09
E1ZES7	Putative uncharacterized protein	CHLNCDRAFT_57872	1.382 ± 0.63	2.135 ± 2.29	2.539 ± 2.60
E1ZKG4	Ferritin	CHLNCDRAFT_58473	1.093 ± 0.24	1.615 ± 0.75	1.502 ± 0.62
A0A087SAG9	MOB kinase activator-like 1	F751_6754	0.875 ± 0.06	1.268 ± 0.28	1.307 ± 0.03
E1Z7W6	Ferredoxin-dependent glutamate synthase, chloroplastic	CHLNCDRAFT_142154	1.167 ± 0.28	1.750 ± 0.97	1.716 ± 0.63
E1Z7X2	Putative uncharacterized protein	CHLNCDRAFT_142161	0.492 ± 0.048	0.560 ± 0.22	0.558 ± 0.03
E1Z7N8	Putative uncharacterized protein	CHLNCDRAFT_142063	1.727 ± 0.26	1.723 ± 0.30	1.656 ± 0.94
E1ZRQ7	Porphobilinogen deaminase, chloroplastic	CHLNCDRAFT_33052	0.775 ± 0.23	1.153 ± 0.30	0.962 ± 0.14
A0A087SAW7	Ribulose bisphosphate carboxylase small chain	F751_5580	1.439 ± 0.19	1.961 ± 0.33	2.192 ± 0.12
E1Z6S6	Putative uncharacterized protein (photosystem II assembly)	CHLNCDRAFT_140330	1.451 ± 0.19	2.346 ± 0.38	2.684 ± 0.55
E1ZBP9	Ferredoxin--NADP reductase	CHLNCDRAFT_35035	1.312 ± 0.12	1.853 ± 0.19	2.121 ± 0.17

E1ZFB3	Expressed protein (Thylakoid lumenal protein)	CHLNCDRAFT_133823	1.046 ± 0.12	1.531 ± 0.37	1.569 ± 0.47
A0A087SP02	Ferredoxin--NADP reductase	F751_1233	1.142 ± 0.24	1.766 ± 0.30	2.235 ± 0.72
E1ZQR2	Putative uncharacterized protein	CHLNCDRAFT_32868	1.139 ± 0.11	2.508 ± 0.76	3.099 ± 0.70
F2YGR0	ATP synthase subunit beta	atpB	0.929 ± 0.06	0.986 ± 0.06	1.118 ± 0.09
E1ZPZ7	Putative uncharacterized protein	CHLNCDRAFT_139312	1.004 ± 0.17	1.781 ± 0.57	1.541 ± 0.11
E1ZG17	Putative uncharacterized protein (Fragment) (peroxidin activity)	CHLNCDRAFT_23497	1.327 ± 0.36	2.203 ± 0.82	2.108 ± 0.73
E1ZB61	Peptidyl-prolyl cis-trans isomerase	CHLNCDRAFT_21564	1.209 ± 0.23	1.798 ± 0.58	1.890 ± 0.30
A0A087SH10	RNA 3'-terminal phosphate cyclase-like protein	F751_1893	1.584 ±0.21	1.095 ± 0.03	2.780 ± 0.68
E1Z823	40S ribosomal protein S18	CHLNCDRAFT_34417	1.080 ± 0.15	1.400 ± 0.54	1.393 ± 0.68
E1ZAY3	Putative uncharacterized protein	CHLNCDRAFT_51198	0.934 ± 0.08	1.106 ±0.34	0.761 ± 0.00
E1ZIH2	Putative uncharacterized protein	CHLNCDRAFT_53148	1.997 ± 0.38	2.266 ± 0.36	2.837 ± 0.35
E1ZCK9	Putative uncharacterized protein (Chloroplastic lipocalin)	CHLNCDRAFT_145104	1.398 ± 0.60	1.773 ± 0.41	2.209 ± 0.30
A0A087SGA4	1-deoxy-D-xylulose-5-phosphate synthase 1, chloroplastic	F751_3770	0.857 ± 0.22	1.149 ± 0.00	1.417 ± 0.10
E1ZN67	Proteasome subunit alpha type	CHLNCDRAFT_26444	1.532 ± 0.02	1.666 ± 0.14	2.599 ± 0.33
E1ZQQ9	Proteasome subunit alpha type	CHLNCDRAFT_27583	0.864 ± 0.49	1.676 ± 0.58	2.030 ± 0.85
E1Z575	Putative uncharacterized protein (Cytosol aminopeptidase)	CHLNCDRAFT_33905	1.493 ± 0.60	1.956 ± 0.50	2.047 ± 0.19
E1ZMK0	Putative uncharacterized protein (metallo peptidase)	CHLNCDRAFT_137495	0.335 ± 0.07	0.835 ± 0.29	2.149 ± 1.37

E1Z5I7	Ubiquitin-60S ribosomal protein L40-2	CHLNCDRAFT_48528	1.038 ± 0.39	1.930 ± 0.55	2.337 ± 0.70
E1ZCR2	Proteasome subunit alpha type	CHLNCDRAFT_48764	0.987 ± 0.20	1.478 ± 0.38	1.741 ± 0.28
A0A087SEY5	Proteasome subunit alpha type-4	F751_5131	1.035 ± 0.24	1.693 ± 0.13	2.566 ± 0.12
E1ZR38	Putative uncharacterized protein	CHLNCDRAFT_139875	1.056 ± 0.18	1.510 ± 0.20	1.684 ± 0.34
E1ZF61	Putative uncharacterized protein	CHLNCDRAFT_133752	0.866 ± 0.15	1.313 ± 0.25	1.116 ± 0.30
E1ZHB2	Putative uncharacterized protein	CHLNCDRAFT_134996	1.575 ± 0.07	1.506 ± 0.09	1.463 ± 0.05
A0A087SLI4	Uncharacterized protein	F751_2175	0.776 ± 0.12	1.867 ± 0.27	2.492 ± 0.40
E1Z626	Putative uncharacterized protein (Pentatricopeptide repeat)	CHLNCDRAFT_140730	3.875 ± 0.15	6.253 ± 0.30	2.559 ± 0.17
E1ZBK2	Elongation factor 1-alpha	CHLNCDRAFT_30705	0.980 ± 0.31	1.430 ± 0.34	1.929 ± 0.55
E1ZE06	S-adenosylmethionine synthase	CHLNCDRAFT_145246	1.162 ± 0.33	1.580 ± 0.43	1.709 ± 0.36
E1ZFK7	Putative uncharacterized protein	CHLNCDRAFT_134242	1.042 ± 0.14	1.674 ± 0.09	1.685 ± 0.17
E1ZG44	Putative uncharacterized protein	CHLNCDRAFT_134507	1.568 ± 0.27	2.584 ± 0.87	2.622 ± 0.52
E1ZK86	Putative uncharacterized protein (Fragment) (WD repeat protein)	CHLNCDRAFT_9620	2.091 ± 0.81	2.418 ± 0.83	2.204 ± 0.49
E1ZM23	Putative uncharacterized protein (WD repeat-containing protein 2)	CHLNCDRAFT_53988	0.417 ± 0.14	0.713 ± 0.22	2.010 ± 0.72
E1ZNS0	Putative uncharacterized protein	CHLNCDRAFT_138794	0.809 ± 0.04	1.743 ± 0.16	1.907 ± 0.12
E1ZPF8	Putative uncharacterized protein	CHLNCDRAFT_139194	3.405 ± 0.19	7.319 ± 0.90	2.584 ± 0.32
E1ZRV4	DNA gyrase subunit A	CHLNCDRAFT_56398	1.181 ± 0.13	2.350 ± 0.64	2.836 ± 0.81
E1ZS12	Putative uncharacterized protein	CHLNCDRAFT_33111	0.882 ± 0.15	1.708 ± 0.55	2.050 ± 0.89
E1ZS63	ATP synthase subunit beta	CHLNCDRAFT_141119	1.193 ± 0.21	1.661 ± 0.45	1.351 ± 0.19
E1ZTT4	Putative uncharacterized protein	CHLNCDRAFT_142513	1.599 ± 0.24	2.436 ± 0.56	2.819 ± 0.51
A0A087SEN2	Uncharacterized protein	F751_5028	1.176 ± 0.07	2.105 ± 0.97	2.126 ± 1.54
A0A087SEW5	Isoleucine-tRNA ligase	F751_5111	1.139 ± 0.31	1.487 ± 0.55	1.672 ± 0.28
A0A087SFN5	WD-40 repeat-containing protein MSI1	F751_2257	1.640 ± 0.01	2.797 ± 0.86	3.225 ± 0.06
A0A087SFR7	DNA repair protein rhp26	F751_2289	1.371 ± 0.01	2.077 ± 0.37	1.982 ± 0.21
A0A087SG16	Uncharacterized protein	F751_2387	1.130 ± 0.04	1.548 ± 0.13	2.020 ± 0.36
A0A087SGR7	NPL4-like protein 2	F751_1800	1.461 ± 0.40	1.945 ± 0.86	1.809 ± 0.21

A0A087SJN6	Uncharacterized protein	F751_4192	1.264 ± 0.01	2.480 ± 0.15	2.413 ± 0.37
A0A087SKQ6	Myb-like protein A	F751_4803	0.786± 0.23	0.887 ± 0.26	0.938 ± 0.11
A0A087SL71	Uncharacterized protein	F751_2693	1.402 ± 0.05	1.945 ± 0.15	1.829 ± 0.18
A0A087SMY2	Uncharacterized protein	F751_5705	0.851 ± 0.19	1.763 ± 0.14	2.142 ± 0.26
A0A087SP60	Uncharacterized protein	F751_5370	1.631 ± 0.40	2.646 ±0.37	2.418 ± 0.48
A0A087SPR1	Putative cadmium/zinc-transporting ATPase HMA1, chloroplastic	F751_5679	1.876 ± 0.24	3.533 ± 0.86	2.296 ± 0.07
A0A087SSB9	Vacuole membrane protein 1-like protein	F751_6382	1.190 ± 0.18	1.811 ± 0.29	2.029 ± 0.14
A0A087SU98	Uncharacterized protein	F751_5740	1.782 ± 0.66	1.602 ± 0.00	1.764 ± 0.29
E1Z215	Putative uncharacterized protein (Glycosyltransferase)	CHLNCDRAFT_132962	0.978 ± 0.09	1.969 ± 0.26	1.752 ± 0.36
E1Z287	Expressed protein	CHLNCDRAFT_133050	1.297	1.550 ± 0.00	1.281 ± 0.06
E1Z2D3	Putative uncharacterized protein (Vacuolar assembling protein VPS41)	CHLNCDRAFT_133107	1.548 ± 0.20	1.953 ± 0.09	2.213 ± 0.62
E1Z2W0	Expressed protein	CHLNCDRAFT_56535	0.929 ± 0.01	1.783 ± 0.48	1.266 ± 0.24
E1Z580	Superoxide dismutase	CHLNCDRAFT_33910	1.000 ± 0.57	1.928 ± 0.89	2.152 ± 1.18
E1Z764	Putative uncharacterized protein (Serine/threonine protein kinase)	CHLNCDRAFT_142454	1.418 ± 0.11	2.119 ± 0.01	2.745 ± 0.25
E1Z7K6	Expressed protein	CHLNCDRAFT_142027	1.255 ± 0.01	1.120 ± 0.27	1.432 ± 0.03
E1ZAY6	Putative uncharacterized protein	CHLNCDRAFT_48679	1.608 ± 0.38	2.459 ± 1.04	2.809 ± 0.65
E1ZAZ1	Putative uncharacterized protein	CHLNCDRAFT_143466	0.789 ± 0.22	1.168 ± 0.30	1.172 ± 0.16
E1ZB67	Putative uncharacterized protein (Inorganic pyrophosphatase)	CHLNCDRAFT_56094	1.150 ± 0.08	1.393 ± 0.17	2.028 ± 0.10
E1ZB69	Putative uncharacterized protein	CHLNCDRAFT_143565	0.786 ± 0.07	0.888 ± 0.00	1.327 ± 0.10
E1ZBE3	Expressed protein	CHLNCDRAFT_57528	0.919 ± 0.80	2.115 ± 0.21	1.978 ± 1.74
E1ZCN4	Putative uncharacterized protein (Pentatricopeptide repeat-containing protein, putative)	CHLNCDRAFT_51958	1.278 ± 0.55	2.530 ± 1.38	2.847 ± 1.47
E1ZDM9	Putative uncharacterized protein RACK1	RACK1	1.150 ± 0.08	1.606 ± 0.40	1.491 ± 0.05

E1ZDW6	Putative uncharacterized protein (DNA topoisomerase)	CHLNCDRAFT_145197	1.571 ± 0.50	1.999 ± 0.71	2.245 ± 0.97
E1ZFI7	Putative uncharacterized protein (methylenetetrahydrofolate dehydrogenase)	CHLNCDRAFT_52464	1.704 ± 0.09	2.088 ± 0.07	2.492 ± 0.13
E1ZKH7	Putative uncharacterized protein	CHLNCDRAFT_136511	0.820 ± 0.14	1.212 ± 0.02	0.828 ± 0.73
E1ZL39	Putative uncharacterized protein	CHLNCDRAFT_136806	1.8756 ± 0.55	1.880 ± 0.54	2.221 ± 0.27
E1ZMB1	Peptidyl-prolyl cis-trans isomerase (Fragment)	CHLNCDRAFT_26227	1.167 ± 0.19	1.712 ± 0.60	1.528 ± 1.34
E1ZQ17	Putative uncharacterized protein	CHLNCDRAFT_139335	1.040 ± 0.41	0.757 ± 0.46	0.755 ± 0.21
E1ZSM3	Putative uncharacterized protein	CHLNCDRAFT_141324	0.446 ± 0.23	0.506 ± 0.14	0.733 ± 0.31
A0A087SI90	DEAD-box ATP-dependent RNA helicase 50	F751_5985	1.194 ± 0.50	2.512 ± 0.72	5.007 ± 1.15
A0A087SQK4	Vacuolar protein sorting-associated protein 11-like protein	F751_6683	1.126 ± 0.72	2.530 ± 1.40	3.180 ± 1.68
E1Z8R4	Putative uncharacterized protein HYDG	HYDG	1.707 ± 0.72	2.161 ± 0.37	2.072 ± 0.02

Table S5: The score, pI, molecular ion, Q1/Q3 transitions, and optimized CE for each peptide obtained in MRM study

Method

<<MS Parameter>>

CID Gas :270 kPa
Conversion Dynode :-10.00 kV
Initial Valve Position :-
--Segment 1 Event 1--
Acquisition Mode :MRM
Polarity :Positive
Start Time :3.550 min
End Time :6.550 min
Compound Name :EGKLEVTK_light

Ch1	(Precursor m/z) 301.85	(Product m/z) 589.35	(Pause Time) 1.0	(Dwell Time) 40.0	(CE) -10.3
Ch2	(Precursor m/z) 301.85	(Product m/z) 476.25	(Pause Time) 1.0	(Dwell Time) 40.0	(CE) -10.3
Ch3	(Precursor m/z) 301.85	(Product m/z) 347.25	(Pause Time) 1.0	(Dwell Time) 40.0	(CE) -10.3

Event Time :0.123 sec
Q1 Resolution :Unit
Q3 Resolution :Unit
Micro Scan Width :0.00 u
Interface Volt. :4.00 kV
DUIS Corona Needle Volt. :4.50 kV
Pause Time / Dwell Time :Set Value of Each Channel (Pause Time & Dwell Time)
DL Bias :Use the Data in the Tuning File
Qarray Bias :Use the Data in the Tuning File
Q1 Prerod Bias :Use the Data in the Tuning File
Q3 Prerod Bias :Use the Data in the Tuning File
--Segment 2 Event 2--
Acquisition Mode :MRM
Polarity :Positive
Start Time :4.557 min
End Time :7.557 min
Compound Name :TQDGGTEVVQAK_light

Ch1	(Precursor m/z) 616.80	(Product m/z) 888.50	(Pause Time) 1.0	(Dwell Time) 30.0	(CE) -24.2
Ch2	(Precursor m/z) 616.80	(Product m/z) 673.40	(Pause Time) 1.0	(Dwell Time) 30.0	(CE) -24.2
Ch3	(Precursor m/z) 616.80	(Product m/z) 445.30	(Pause Time) 1.0	(Dwell Time) 30.0	(CE) -24.2
Ch4	(Precursor m/z) 616.80	(Product m/z) 346.20	(Pause Time) 1.0	(Dwell Time) 30.0	(CE) -24.2

Event Time :0.124 sec
Q1 Resolution :Unit
Q3 Resolution :Unit
Micro Scan Width :0.00 u
Interface Volt. :4.00 kV
DUIS Corona Needle Volt. :4.50 kV
Pause Time / Dwell Time :Set Value of Each Channel (Pause Time & Dwell Time)
DL Bias :Use the Data in the Tuning File
Qarray Bias :Use the Data in the Tuning File
Q1 Prerod Bias :Use the Data in the Tuning File
Q3 Prerod Bias :Use the Data in the Tuning File
--Segment 3 Event 3--
Acquisition Mode :MRM
Polarity :Positive
Start Time :6.179 min
End Time :9.179 min
Compound Name :LVEGLGGSK_light

Ch1	(Precursor m/z) 430.25	(Product m/z) 518.30	(Pause Time) 1.0	(Dwell Time) 4.0	(CE) -16.7
Ch2	(Precursor m/z) 430.25	(Product m/z) 461.25	(Pause Time) 1.0	(Dwell Time) 4.0	(CE) -16.7
Ch3	(Precursor m/z) 430.25	(Product m/z) 348.20	(Pause Time) 1.0	(Dwell Time) 4.0	(CE) -16.7
Ch4	(Precursor m/z) 430.25	(Product m/z) 291.15	(Pause Time) 1.0	(Dwell Time) 4.0	(CE) -16.7
Ch5	(Precursor m/z) 430.25	(Product m/z) 234.15	(Pause Time) 1.0	(Dwell Time) 4.0	(CE) -16.7

Event Time :0.025 sec
Q1 Resolution :Unit
Q3 Resolution :Unit
Micro Scan Width :0.00 u
Interface Volt. :4.00 kV
DUIS Corona Needle Volt. :4.50 kV
Pause Time / Dwell Time :Set Value of Each Channel (Pause Time & Dwell Time)
DL Bias :Use the Data in the Tuning File
Qarray Bias :Use the Data in the Tuning File
Q1 Prerod Bias :Use the Data in the Tuning File
Q3 Prerod Bias :Use the Data in the Tuning File
--Segment 4 Event 4--
Acquisition Mode :MRM
Polarity :Positive
Start Time :6.539 min
End Time :9.539 min
Compound Name :ALQNTVLK_light

Ch1	(Precursor m/z) 443.75	(Product m/z) 574.35	(Pause Time) 1.0	(Dwell Time) 4.0	(CE) -17.2
Ch2	(Precursor m/z) 443.75	(Product m/z) 460.30	(Pause Time) 1.0	(Dwell Time) 4.0	(CE) -17.2
Ch3	(Precursor m/z) 443.75	(Product m/z) 359.25	(Pause Time) 1.0	(Dwell Time) 4.0	(CE) -17.2
Ch4	(Precursor m/z) 443.75	(Product m/z) 260.20	(Pause Time) 1.0	(Dwell Time) 4.0	(CE) -17.2

Event Time :0.020 sec
Q1 Resolution :Unit

Q3 Resolution :Unit
Micro Scan Width :0.00 u
Interface Volt. :4.00 kV
DUIS Corona Needle Volt. :4.50 kV
Pause Time / Dwell Time :Set Value of Each Channel (Pause Time & Dwell Time)
DL Bias :Use the Data in the Tuning File
Qarray Bias :Use the Data in the Tuning File
Q1 Prerod Bias :Use the Data in the Tuning File
Q3 Prerod Bias :Use the Data in the Tuning File
--Segment 5 Event 5--
Acquisition Mode :MRM
Polarity :Positive
Start Time :6.569 min
End Time :9.569 min
Compound Name :DC[+57.0]AYGLAR_light
Ch1 (Precursor m/z) 463.20 (Product m/z) 579.30 (Pause Time) 1.0 (Dwell Time) 4.0 (CE) -18.0
Ch2 (Precursor m/z) 463.20 (Product m/z) 416.25 (Pause Time) 1.0 (Dwell Time) 4.0 (CE) -18.0
Ch3 (Precursor m/z) 463.20 (Product m/z) 359.25 (Pause Time) 1.0 (Dwell Time) 4.0 (CE) -18.0
Ch4 (Precursor m/z) 463.20 (Product m/z) 246.15 (Pause Time) 1.0 (Dwell Time) 4.0 (CE) -18.0
Event Time :0.020 sec
Q1 Resolution :Unit
Q3 Resolution :Unit
Micro Scan Width :0.00 u
Interface Volt. :4.00 kV
DUIS Corona Needle Volt. :4.50 kV
Pause Time / Dwell Time :Set Value of Each Channel (Pause Time & Dwell Time)
DL Bias :Use the Data in the Tuning File
Qarray Bias :Use the Data in the Tuning File
Q1 Prerod Bias :Use the Data in the Tuning File
Q3 Prerod Bias :Use the Data in the Tuning File
--Segment 6 Event 6--
Acquisition Mode :MRM
Polarity :Positive
Start Time :6.571 min
End Time :9.571 min
Compound Name :IIYGGSVTAK_light
Ch1 (Precursor m/z) 504.80 (Product m/z) 390.25 (Pause Time) 1.0 (Dwell Time) 10.0 (CE) -19.7
Ch2 (Precursor m/z) 504.80 (Product m/z) 447.25 (Pause Time) 1.0 (Dwell Time) 10.0 (CE) -19.7
Ch3 (Precursor m/z) 504.80 (Product m/z) 504.30 (Pause Time) 1.0 (Dwell Time) 10.0 (CE) -19.7
Ch4 (Precursor m/z) 504.80 (Product m/z) 591.30 (Pause Time) 1.0 (Dwell Time) 10.0 (CE) -19.7
Ch5 (Precursor m/z) 504.80 (Product m/z) 345.70 (Pause Time) 1.0 (Dwell Time) 10.0 (CE) -19.7
Ch6 (Precursor m/z) 504.80 (Product m/z) 431.75 (Pause Time) 1.0 (Dwell Time) 10.0 (CE) -19.7
Event Time :0.066 sec
Q1 Resolution :Unit
Q3 Resolution :Unit
Micro Scan Width :0.00 u
Interface Volt. :4.00 kV
DUIS Corona Needle Volt. :4.50 kV
Pause Time / Dwell Time :Set Value of Each Channel (Pause Time & Dwell Time)
DL Bias :Use the Data in the Tuning File
Qarray Bias :Use the Data in the Tuning File
Q1 Prerod Bias :Use the Data in the Tuning File
Q3 Prerod Bias :Use the Data in the Tuning File
--Segment 7 Event 7--
Acquisition Mode :MRM
Polarity :Positive
Start Time :6.607 min
End Time :9.607 min
Compound Name :GILAM[+16.0]DESNATC[+57.0]GK_light
Ch1 (Precursor m/z) 741.85 (Product m/z) 1128.45 (Pause Time) 1.0 (Dwell Time) 10.0 (CE) -29.2
Ch2 (Precursor m/z) 741.85 (Product m/z) 981.40 (Pause Time) 1.0 (Dwell Time) 10.0 (CE) -29.2
Ch3 (Precursor m/z) 741.85 (Product m/z) 737.30 (Pause Time) 1.0 (Dwell Time) 10.0 (CE) -29.2
Ch4 (Precursor m/z) 741.85 (Product m/z) 465.20 (Pause Time) 1.0 (Dwell Time) 10.0 (CE) -29.2
Ch5 (Precursor m/z) 741.85 (Product m/z) 364.15 (Pause Time) 1.0 (Dwell Time) 10.0 (CE) -29.2
Ch6 (Precursor m/z) 741.85 (Product m/z) 204.15 (Pause Time) 1.0 (Dwell Time) 10.0 (CE) -29.2
Ch7 (Precursor m/z) 741.85 (Product m/z) 600.25 (Pause Time) 1.0 (Dwell Time) 10.0 (CE) -29.2
Ch8 (Precursor m/z) 741.85 (Product m/z) 564.70 (Pause Time) 1.0 (Dwell Time) 10.0 (CE) -29.2
Event Time :0.088 sec
Q1 Resolution :Unit
Q3 Resolution :Unit
Micro Scan Width :0.00 u
Interface Volt. :4.00 kV
DUIS Corona Needle Volt. :4.50 kV
Pause Time / Dwell Time :Set Value of Each Channel (Pause Time & Dwell Time)
DL Bias :Use the Data in the Tuning File
Qarray Bias :Use the Data in the Tuning File
Q1 Prerod Bias :Use the Data in the Tuning File
Q3 Prerod Bias :Use the Data in the Tuning File
--Segment 8 Event 8--
Acquisition Mode :MRM
Polarity :Positive

Start Time :6.702 min
End Time :9.702 min
Compound Name :LVTDLTK_light

Ch1	(Precursor m/z) 395.25	(Product m/z) 676.40	(Pause Time) 1.0	(Dwell Time) 3.0	(CE) -15.3
Ch2	(Precursor m/z) 395.25	(Product m/z) 577.30	(Pause Time) 1.0	(Dwell Time) 3.0	(CE) -15.3
Ch3	(Precursor m/z) 395.25	(Product m/z) 361.25	(Pause Time) 1.0	(Dwell Time) 3.0	(CE) -15.3
Ch4	(Precursor m/z) 395.25	(Product m/z) 248.15	(Pause Time) 1.0	(Dwell Time) 3.0	(CE) -15.3
Ch5	(Precursor m/z) 395.25	(Product m/z) 147.10	(Pause Time) 1.0	(Dwell Time) 3.0	(CE) -15.3

Event Time :0.020 sec
Q1 Resolution :Unit
Q3 Resolution :Unit
Micro Scan Width :0.00 u
Interface Volt. :4.00 kV
DUIS Corona Needle Volt. :4.50 kV
Pause Time / Dwell Time :Set Value of Each Channel (Pause Time & Dwell Time)
DL Bias :Use the Data in the Tuning File
Qarray Bias :Use the Data in the Tuning File
Q1 Prerod Bias :Use the Data in the Tuning File
Q3 Prerod Bias :Use the Data in the Tuning File
--Segment 9 Event 9--
Acquisition Mode :MRM
Polarity :Positive
Start Time :6.717 min
End Time :9.717 min
Compound Name :VIITAPAK_light

Ch1	(Precursor m/z) 406.75	(Product m/z) 487.30	(Pause Time) 1.0	(Dwell Time) 6.0	(CE) -15.8
Ch2	(Precursor m/z) 406.75	(Product m/z) 386.25	(Pause Time) 1.0	(Dwell Time) 6.0	(CE) -15.8
Ch3	(Precursor m/z) 406.75	(Product m/z) 315.20	(Pause Time) 1.0	(Dwell Time) 6.0	(CE) -15.8

Event Time :0.021 sec
Q1 Resolution :Unit
Q3 Resolution :Unit
Micro Scan Width :0.00 u
Interface Volt. :4.00 kV
DUIS Corona Needle Volt. :4.50 kV
Pause Time / Dwell Time :Set Value of Each Channel (Pause Time & Dwell Time)
DL Bias :Use the Data in the Tuning File
Qarray Bias :Use the Data in the Tuning File
Q1 Prerod Bias :Use the Data in the Tuning File
Q3 Prerod Bias :Use the Data in the Tuning File
--Segment 10 Event 10--
Acquisition Mode :MRM
Polarity :Positive
Start Time :6.774 min
End Time :9.774 min
Compound Name :GAC[+57.0]LLPK_light

Ch1	(Precursor m/z) 379.70	(Product m/z) 630.35	(Pause Time) 1.0	(Dwell Time) 3.0	(CE) -14.7
Ch2	(Precursor m/z) 379.70	(Product m/z) 470.35	(Pause Time) 1.0	(Dwell Time) 3.0	(CE) -14.7
Ch3	(Precursor m/z) 379.70	(Product m/z) 357.25	(Pause Time) 1.0	(Dwell Time) 3.0	(CE) -14.7
Ch4	(Precursor m/z) 379.70	(Product m/z) 244.15	(Pause Time) 1.0	(Dwell Time) 3.0	(CE) -14.7
Ch5	(Precursor m/z) 379.70	(Product m/z) 315.70	(Pause Time) 1.0	(Dwell Time) 3.0	(CE) -14.7

Event Time :0.020 sec
Q1 Resolution :Unit
Q3 Resolution :Unit
Micro Scan Width :0.00 u
Interface Volt. :4.00 kV
DUIS Corona Needle Volt. :4.50 kV
Pause Time / Dwell Time :Set Value of Each Channel (Pause Time & Dwell Time)
DL Bias :Use the Data in the Tuning File
Qarray Bias :Use the Data in the Tuning File
Q1 Prerod Bias :Use the Data in the Tuning File
Q3 Prerod Bias :Use the Data in the Tuning File
--Segment 11 Event 11--
Acquisition Mode :MRM
Polarity :Positive
Start Time :7.040 min
End Time :10.040 min
Compound Name :AEFVEVTK_light

Ch1	(Precursor m/z) 461.75	(Product m/z) 722.40	(Pause Time) 1.0	(Dwell Time) 3.0	(CE) -18.0
Ch2	(Precursor m/z) 461.75	(Product m/z) 575.35	(Pause Time) 1.0	(Dwell Time) 3.0	(CE) -18.0
Ch3	(Precursor m/z) 461.75	(Product m/z) 476.25	(Pause Time) 1.0	(Dwell Time) 3.0	(CE) -18.0
Ch4	(Precursor m/z) 461.75	(Product m/z) 347.25	(Pause Time) 1.0	(Dwell Time) 3.0	(CE) -18.0
Ch5	(Precursor m/z) 461.75	(Product m/z) 248.15	(Pause Time) 1.0	(Dwell Time) 3.0	(CE) -18.0

Event Time :0.020 sec
Q1 Resolution :Unit
Q3 Resolution :Unit
Micro Scan Width :0.00 u
Interface Volt. :4.00 kV
DUIS Corona Needle Volt. :4.50 kV
Pause Time / Dwell Time :Set Value of Each Channel (Pause Time & Dwell Time)
DL Bias :Use the Data in the Tuning File
Qarray Bias :Use the Data in the Tuning File

Q1 Prerod Bias :Use the Data in the Tuning File
Q3 Prerod Bias :Use the Data in the Tuning File
--Segment 12 Event 12--
Acquisition Mode :MRM
Polarity :Positive
Start Time :7.834 min
End Time :10.834 min
Compound Name :GVFTNVTSPSTK_light
Ch1 (Precursor m/z) 619.30 (Product m/z) 833.45 (Pause Time) 1.0 (Dwell Time) 3.0 (CE) -24.3
Ch2 (Precursor m/z) 619.30 (Product m/z) 719.40 (Pause Time) 1.0 (Dwell Time) 3.0 (CE) -24.3
Ch3 (Precursor m/z) 619.30 (Product m/z) 620.30 (Pause Time) 1.0 (Dwell Time) 3.0 (CE) -24.3
Ch4 (Precursor m/z) 619.30 (Product m/z) 519.30 (Pause Time) 1.0 (Dwell Time) 3.0 (CE) -24.3
Ch5 (Precursor m/z) 619.30 (Product m/z) 432.25 (Pause Time) 1.0 (Dwell Time) 3.0 (CE) -24.3
Event Time :0.020 sec
Q1 Resolution :Unit
Q3 Resolution :Unit
Micro Scan Width :0.00 u
Interface Volt. :4.00 kV
DUIS Corona Needle Volt. :4.50 kV
Pause Time / Dwell Time :Set Value of Each Channel (Pause Time & Dwell Time)
DL Bias :Use the Data in the Tuning File
Qarray Bias :Use the Data in the Tuning File
Q1 Prerod Bias :Use the Data in the Tuning File
Q3 Prerod Bias :Use the Data in the Tuning File
--Segment 13 Event 13--
Acquisition Mode :MRM
Polarity :Positive
Start Time :8.112 min
End Time :11.112 min
Compound Name :HLVDEPQNLIK_light
Ch1 (Precursor m/z) 435.90 (Product m/z) 712.45 (Pause Time) 1.0 (Dwell Time) 3.0 (CE) -15.3
Ch2 (Precursor m/z) 435.90 (Product m/z) 487.30 (Pause Time) 1.0 (Dwell Time) 3.0 (CE) -15.3
Ch3 (Precursor m/z) 435.90 (Product m/z) 260.20 (Pause Time) 1.0 (Dwell Time) 3.0 (CE) -15.3
Ch4 (Precursor m/z) 435.90 (Product m/z) 147.10 (Pause Time) 1.0 (Dwell Time) 3.0 (CE) -15.3
Ch5 (Precursor m/z) 435.90 (Product m/z) 356.70 (Pause Time) 1.0 (Dwell Time) 3.0 (CE) -15.3
Event Time :0.020 sec
Q1 Resolution :Unit
Q3 Resolution :Unit
Micro Scan Width :0.00 u
Interface Volt. :4.00 kV
DUIS Corona Needle Volt. :4.50 kV
Pause Time / Dwell Time :Set Value of Each Channel (Pause Time & Dwell Time)
DL Bias :Use the Data in the Tuning File
Qarray Bias :Use the Data in the Tuning File
Q1 Prerod Bias :Use the Data in the Tuning File
Q3 Prerod Bias :Use the Data in the Tuning File
--Segment 14 Event 14--
Acquisition Mode :MRM
Polarity :Positive
Start Time :8.163 min
End Time :11.163 min
Compound Name :FVC[+57.0]IVDDSK_light
Ch1 (Precursor m/z) 541.75 (Product m/z) 676.35 (Pause Time) 1.0 (Dwell Time) 10.0 (CE) -21.2
Ch2 (Precursor m/z) 541.75 (Product m/z) 563.25 (Pause Time) 1.0 (Dwell Time) 10.0 (CE) -21.2
Ch3 (Precursor m/z) 541.75 (Product m/z) 464.20 (Pause Time) 1.0 (Dwell Time) 10.0 (CE) -21.2
Ch4 (Precursor m/z) 541.75 (Product m/z) 349.15 (Pause Time) 1.0 (Dwell Time) 10.0 (CE) -21.2
Ch5 (Precursor m/z) 541.75 (Product m/z) 234.15 (Pause Time) 1.0 (Dwell Time) 10.0 (CE) -21.2
Event Time :0.055 sec
Q1 Resolution :Unit
Q3 Resolution :Unit
Micro Scan Width :0.00 u
Interface Volt. :4.00 kV
DUIS Corona Needle Volt. :4.50 kV
Pause Time / Dwell Time :Set Value of Each Channel (Pause Time & Dwell Time)
DL Bias :Use the Data in the Tuning File
Qarray Bias :Use the Data in the Tuning File
Q1 Prerod Bias :Use the Data in the Tuning File
Q3 Prerod Bias :Use the Data in the Tuning File
--Segment 15 Event 15--
Acquisition Mode :MRM
Polarity :Positive
Start Time :8.348 min
End Time :11.348 min
Compound Name :HIIGESDEFIADK_light
Ch1 (Precursor m/z) 491.90 (Product m/z) 364.25 (Pause Time) 1.0 (Dwell Time) 10.0 (CE) -17.4
Ch2 (Precursor m/z) 491.90 (Product m/z) 421.25 (Pause Time) 1.0 (Dwell Time) 10.0 (CE) -17.4
Ch3 (Precursor m/z) 491.90 (Product m/z) 550.30 (Pause Time) 1.0 (Dwell Time) 10.0 (CE) -17.4
Ch4 (Precursor m/z) 491.90 (Product m/z) 637.35 (Pause Time) 1.0 (Dwell Time) 10.0 (CE) -17.4
Ch5 (Precursor m/z) 491.90 (Product m/z) 752.35 (Pause Time) 1.0 (Dwell Time) 10.0 (CE) -17.4
Ch6 (Precursor m/z) 491.90 (Product m/z) 881.40 (Pause Time) 1.0 (Dwell Time) 10.0 (CE) -17.4
Ch7 (Precursor m/z) 491.90 (Product m/z) 1028.45 (Pause Time) 1.0 (Dwell Time) 10.0 (CE) -17.4

Ch8 (Precursor m/z) 491.90 (Product m/z) 319.15 (Pause Time) 1.0 (Dwell Time) 10.0 (CE) -17.4
Ch9 (Precursor m/z) 491.90 (Product m/z) 376.70 (Pause Time) 1.0 (Dwell Time) 10.0 (CE) -17.4
Ch10 (Precursor m/z) 491.90 (Product m/z) 441.20 (Pause Time) 1.0 (Dwell Time) 10.0 (CE) -17.4
Ch11 (Precursor m/z) 491.90 (Product m/z) 514.75 (Pause Time) 1.0 (Dwell Time) 10.0 (CE) -17.4
Ch12 (Precursor m/z) 491.90 (Product m/z) 571.30 (Pause Time) 1.0 (Dwell Time) 10.0 (CE) -17.4
Event Time :0.132 sec
Q1 Resolution :Unit
Q3 Resolution :Unit
Micro Scan Width :0.00 u
Interface Volt. :4.00 kV
DUIS Corona Needle Volt. :4.50 kV
Pause Time / Dwell Time :Set Value of Each Channel (Pause Time & Dwell Time)
DL Bias :Use the Data in the Tuning File
Qarray Bias :Use the Data in the Tuning File
Q1 Prerod Bias :Use the Data in the Tuning File
Q3 Prerod Bias :Use the Data in the Tuning File
--Segment 16 Event 16--
Acquisition Mode :MRM
Polarity :Positive
Start Time :8.429 min
End Time :11.429 min
Compound Name :DQAAAGMGIYGPR_light
Ch1 (Precursor m/z) 653.80 (Product m/z) 992.50 (Pause Time) 1.0 (Dwell Time) 3.0 (CE) -25.6
Ch2 (Precursor m/z) 653.80 (Product m/z) 921.45 (Pause Time) 1.0 (Dwell Time) 3.0 (CE) -25.6
Ch3 (Precursor m/z) 653.80 (Product m/z) 850.40 (Pause Time) 1.0 (Dwell Time) 3.0 (CE) -25.6
Ch4 (Precursor m/z) 653.80 (Product m/z) 662.35 (Pause Time) 1.0 (Dwell Time) 3.0 (CE) -25.6
Ch5 (Precursor m/z) 653.80 (Product m/z) 329.20 (Pause Time) 1.0 (Dwell Time) 3.0 (CE) -25.6
Event Time :0.020 sec
Q1 Resolution :Unit
Q3 Resolution :Unit
Micro Scan Width :0.00 u
Interface Volt. :4.00 kV
DUIS Corona Needle Volt. :4.50 kV
Pause Time / Dwell Time :Set Value of Each Channel (Pause Time & Dwell Time)
DL Bias :Use the Data in the Tuning File
Qarray Bias :Use the Data in the Tuning File
Q1 Prerod Bias :Use the Data in the Tuning File
Q3 Prerod Bias :Use the Data in the Tuning File
--Segment 17 Event 17--
Acquisition Mode :MRM
Polarity :Positive
Start Time :8.606 min
End Time :11.606 min
Compound Name :FYGEITQQM[+16.0]LK_light
Ch1 (Precursor m/z) 687.35 (Product m/z) 877.50 (Pause Time) 1.0 (Dwell Time) 3.0 (CE) -27.0
Ch2 (Precursor m/z) 687.35 (Product m/z) 764.40 (Pause Time) 1.0 (Dwell Time) 3.0 (CE) -27.0
Ch3 (Precursor m/z) 687.35 (Product m/z) 663.35 (Pause Time) 1.0 (Dwell Time) 3.0 (CE) -27.0
Ch4 (Precursor m/z) 687.35 (Product m/z) 535.30 (Pause Time) 1.0 (Dwell Time) 3.0 (CE) -27.0
Ch5 (Precursor m/z) 687.35 (Product m/z) 407.25 (Pause Time) 1.0 (Dwell Time) 3.0 (CE) -27.0
Event Time :0.020 sec
Q1 Resolution :Unit
Q3 Resolution :Unit
Micro Scan Width :0.00 u
Interface Volt. :4.00 kV
DUIS Corona Needle Volt. :4.50 kV
Pause Time / Dwell Time :Set Value of Each Channel (Pause Time & Dwell Time)
DL Bias :Use the Data in the Tuning File
Qarray Bias :Use the Data in the Tuning File
Q1 Prerod Bias :Use the Data in the Tuning File
Q3 Prerod Bias :Use the Data in the Tuning File
--Segment 18 Event 18--
Acquisition Mode :MRM
Polarity :Positive
Start Time :8.707 min
End Time :11.707 min
Compound Name :KPDFDAYIDPQK_light
Ch1 (Precursor m/z) 479.55 (Product m/z) 763.40 (Pause Time) 1.0 (Dwell Time) 3.0 (CE) -16.9
Ch2 (Precursor m/z) 479.55 (Product m/z) 600.35 (Pause Time) 1.0 (Dwell Time) 3.0 (CE) -16.9
Ch3 (Precursor m/z) 479.55 (Product m/z) 487.25 (Pause Time) 1.0 (Dwell Time) 3.0 (CE) -16.9
Ch4 (Precursor m/z) 479.55 (Product m/z) 372.20 (Pause Time) 1.0 (Dwell Time) 3.0 (CE) -16.9
Ch5 (Precursor m/z) 479.55 (Product m/z) 300.65 (Pause Time) 1.0 (Dwell Time) 3.0 (CE) -16.9
Event Time :0.020 sec
Q1 Resolution :Unit
Q3 Resolution :Unit
Micro Scan Width :0.00 u
Interface Volt. :4.00 kV
DUIS Corona Needle Volt. :4.50 kV
Pause Time / Dwell Time :Set Value of Each Channel (Pause Time & Dwell Time)
DL Bias :Use the Data in the Tuning File
Qarray Bias :Use the Data in the Tuning File
Q1 Prerod Bias :Use the Data in the Tuning File

Q3 Prerod Bias :Use the Data in the Tuning File
--Segment 19 Event 19--
Acquisition Mode :MRM
Polarity :Positive
Start Time :8.812 min
End Time :11.812 min
Compound Name :NPSGEDINALEQHIK_light
Ch1 (Precursor m/z) 555.60 (Product m/z) 654.35 (Pause Time) 1.0 (Dwell Time) 10.0 (CE) -19.7
Ch2 (Precursor m/z) 555.60 (Product m/z) 525.30 (Pause Time) 1.0 (Dwell Time) 10.0 (CE) -19.7
Ch3 (Precursor m/z) 555.60 (Product m/z) 397.25 (Pause Time) 1.0 (Dwell Time) 10.0 (CE) -19.7
Ch4 (Precursor m/z) 555.60 (Product m/z) 655.35 (Pause Time) 1.0 (Dwell Time) 10.0 (CE) -19.7
Ch5 (Precursor m/z) 555.60 (Product m/z) 476.75 (Pause Time) 1.0 (Dwell Time) 10.0 (CE) -19.7
Ch6 (Precursor m/z) 555.60 (Product m/z) 384.20 (Pause Time) 1.0 (Dwell Time) 10.0 (CE) -19.7
Event Time :0.066 sec
Q1 Resolution :Unit
Q3 Resolution :Unit
Micro Scan Width :0.00 u
Interface Volt. :4.00 kV
DUIS Corona Needle Volt. :4.50 kV
Pause Time / Dwell Time :Set Value of Each Channel (Pause Time & Dwell Time)
DL Bias :Use the Data in the Tuning File
Qarray Bias :Use the Data in the Tuning File
Q1 Prerod Bias :Use the Data in the Tuning File
Q3 Prerod Bias :Use the Data in the Tuning File
--Segment 20 Event 20--
Acquisition Mode :MRM
Polarity :Positive
Start Time :8.871 min
End Time :11.871 min
Compound Name :VATPEQAQEVHDAVR_light
Ch1 (Precursor m/z) 550.60 (Product m/z) 498.25 (Pause Time) 1.0 (Dwell Time) 10.0 (CE) -19.5
Ch2 (Precursor m/z) 550.60 (Product m/z) 626.30 (Pause Time) 1.0 (Dwell Time) 10.0 (CE) -19.5
Ch3 (Precursor m/z) 550.60 (Product m/z) 697.35 (Pause Time) 1.0 (Dwell Time) 10.0 (CE) -19.5
Ch4 (Precursor m/z) 550.60 (Product m/z) 825.40 (Pause Time) 1.0 (Dwell Time) 10.0 (CE) -19.5
Ch5 (Precursor m/z) 550.60 (Product m/z) 954.45 (Pause Time) 1.0 (Dwell Time) 10.0 (CE) -19.5
Ch6 (Precursor m/z) 550.60 (Product m/z) 349.20 (Pause Time) 1.0 (Dwell Time) 10.0 (CE) -19.5
Ch7 (Precursor m/z) 550.60 (Product m/z) 413.20 (Pause Time) 1.0 (Dwell Time) 10.0 (CE) -19.5
Ch8 (Precursor m/z) 550.60 (Product m/z) 527.25 (Pause Time) 1.0 (Dwell Time) 10.0 (CE) -19.5
Ch9 (Precursor m/z) 550.60 (Product m/z) 653.30 (Pause Time) 1.0 (Dwell Time) 10.0 (CE) -19.5
Ch10 (Precursor m/z) 550.60 (Product m/z) 688.85 (Pause Time) 1.0 (Dwell Time) 10.0 (CE) -19.5
Event Time :0.110 sec
Q1 Resolution :Unit
Q3 Resolution :Unit
Micro Scan Width :0.00 u
Interface Volt. :4.00 kV
DUIS Corona Needle Volt. :4.50 kV
Pause Time / Dwell Time :Set Value of Each Channel (Pause Time & Dwell Time)
DL Bias :Use the Data in the Tuning File
Qarray Bias :Use the Data in the Tuning File
Q1 Prerod Bias :Use the Data in the Tuning File
Q3 Prerod Bias :Use the Data in the Tuning File
--Segment 21 Event 21--
Acquisition Mode :MRM
Polarity :Positive
Start Time :8.897 min
End Time :11.897 min
Compound Name :GYAGEDQLGEALK_light
Ch1 (Precursor m/z) 675.85 (Product m/z) 1059.55 (Pause Time) 1.0 (Dwell Time) 10.0 (CE) -26.5
Ch2 (Precursor m/z) 675.85 (Product m/z) 873.45 (Pause Time) 1.0 (Dwell Time) 10.0 (CE) -26.5
Ch3 (Precursor m/z) 675.85 (Product m/z) 630.40 (Pause Time) 1.0 (Dwell Time) 10.0 (CE) -26.5
Ch4 (Precursor m/z) 675.85 (Product m/z) 517.30 (Pause Time) 1.0 (Dwell Time) 10.0 (CE) -26.5
Ch5 (Precursor m/z) 675.85 (Product m/z) 331.25 (Pause Time) 1.0 (Dwell Time) 10.0 (CE) -26.5
Event Time :0.055 sec
Q1 Resolution :Unit
Q3 Resolution :Unit
Micro Scan Width :0.00 u
Interface Volt. :4.00 kV
DUIS Corona Needle Volt. :4.50 kV
Pause Time / Dwell Time :Set Value of Each Channel (Pause Time & Dwell Time)
DL Bias :Use the Data in the Tuning File
Qarray Bias :Use the Data in the Tuning File
Q1 Prerod Bias :Use the Data in the Tuning File
Q3 Prerod Bias :Use the Data in the Tuning File
--Segment 22 Event 22--
Acquisition Mode :MRM
Polarity :Positive
Start Time :9.024 min
End Time :12.024 min
Compound Name :AAALNIVPTTTGAAK_light
Ch1 (Precursor m/z) 699.90 (Product m/z) 1072.60 (Pause Time) 1.0 (Dwell Time) 3.0 (CE) -27.5
Ch2 (Precursor m/z) 699.90 (Product m/z) 958.55 (Pause Time) 1.0 (Dwell Time) 3.0 (CE) -27.5

Ch3 (Precursor m/z) 699.90 (Product m/z) 845.45 (Pause Time) 1.0 (Dwell Time) 3.0 (CE) -27.5
Ch4 (Precursor m/z) 699.90 (Product m/z) 746.40 (Pause Time) 1.0 (Dwell Time) 3.0 (CE) -27.5
Ch5 (Precursor m/z) 699.90 (Product m/z) 346.20 (Pause Time) 1.0 (Dwell Time) 3.0 (CE) -27.5
Event Time :0.020 sec
Q1 Resolution :Unit
Q3 Resolution :Unit
Micro Scan Width :0.00 u
Interface Volt. :4.00 kV
DUIS Corona Needle Volt. :4.50 kV
Pause Time / Dwell Time :Set Value of Each Channel (Pause Time & Dwell Time)
DL Bias :Use the Data in the Tuning File
Qarray Bias :Use the Data in the Tuning File
Q1 Prerod Bias :Use the Data in the Tuning File
Q3 Prerod Bias :Use the Data in the Tuning File
--Segment 23 Event 23--
Acquisition Mode :MRM
Polarity :Positive
Start Time :9.239 min
End Time :12.239 min
Compound Name :LISYYLGEK_light
Ch1 (Precursor m/z) 543.30 (Product m/z) 772.40 (Pause Time) 1.0 (Dwell Time) 4.0 (CE) -21.2
Ch2 (Precursor m/z) 543.30 (Product m/z) 609.30 (Pause Time) 1.0 (Dwell Time) 4.0 (CE) -21.2
Ch3 (Precursor m/z) 543.30 (Product m/z) 446.25 (Pause Time) 1.0 (Dwell Time) 4.0 (CE) -21.2
Ch4 (Precursor m/z) 543.30 (Product m/z) 333.20 (Pause Time) 1.0 (Dwell Time) 4.0 (CE) -21.2
Event Time :0.020 sec
Q1 Resolution :Unit
Q3 Resolution :Unit
Micro Scan Width :0.00 u
Interface Volt. :4.00 kV
DUIS Corona Needle Volt. :4.50 kV
Pause Time / Dwell Time :Set Value of Each Channel (Pause Time & Dwell Time)
DL Bias :Use the Data in the Tuning File
Qarray Bias :Use the Data in the Tuning File
Q1 Prerod Bias :Use the Data in the Tuning File
Q3 Prerod Bias :Use the Data in the Tuning File
--Segment 24 Event 24--
Acquisition Mode :MRM
Polarity :Positive
Start Time :9.260 min
End Time :12.260 min
Compound Name :TGLPYIWHSK_light
Ch1 (Precursor m/z) 401.20 (Product m/z) 833.45 (Pause Time) 1.0 (Dwell Time) 10.0 (CE) -14.0
Ch2 (Precursor m/z) 401.20 (Product m/z) 670.35 (Pause Time) 1.0 (Dwell Time) 10.0 (CE) -14.0
Ch3 (Precursor m/z) 401.20 (Product m/z) 557.30 (Pause Time) 1.0 (Dwell Time) 10.0 (CE) -14.0
Ch4 (Precursor m/z) 401.20 (Product m/z) 465.75 (Pause Time) 1.0 (Dwell Time) 10.0 (CE) -14.0
Ch5 (Precursor m/z) 401.20 (Product m/z) 417.20 (Pause Time) 1.0 (Dwell Time) 10.0 (CE) -14.0
Event Time :0.055 sec
Q1 Resolution :Unit
Q3 Resolution :Unit
Micro Scan Width :0.00 u
Interface Volt. :4.00 kV
DUIS Corona Needle Volt. :4.50 kV
Pause Time / Dwell Time :Set Value of Each Channel (Pause Time & Dwell Time)
DL Bias :Use the Data in the Tuning File
Qarray Bias :Use the Data in the Tuning File
Q1 Prerod Bias :Use the Data in the Tuning File
Q3 Prerod Bias :Use the Data in the Tuning File
--Segment 25 Event 25--
Acquisition Mode :MRM
Polarity :Positive
Start Time :10.279 min
End Time :13.279 min
Compound Name :AGIQLSDTFVK_light
Ch1 (Precursor m/z) 589.80 (Product m/z) 937.50 (Pause Time) 1.0 (Dwell Time) 5.0 (CE) -23.1
Ch2 (Precursor m/z) 589.80 (Product m/z) 809.45 (Pause Time) 1.0 (Dwell Time) 5.0 (CE) -23.1
Ch3 (Precursor m/z) 589.80 (Product m/z) 696.35 (Pause Time) 1.0 (Dwell Time) 5.0 (CE) -23.1
Ch4 (Precursor m/z) 589.80 (Product m/z) 609.30 (Pause Time) 1.0 (Dwell Time) 5.0 (CE) -23.1
Ch5 (Precursor m/z) 589.80 (Product m/z) 494.30 (Pause Time) 1.0 (Dwell Time) 5.0 (CE) -23.1
Event Time :0.030 sec
Q1 Resolution :Unit
Q3 Resolution :Unit
Micro Scan Width :0.00 u
Interface Volt. :4.00 kV
DUIS Corona Needle Volt. :4.50 kV
Pause Time / Dwell Time :Set Value of Each Channel (Pause Time & Dwell Time)
DL Bias :Use the Data in the Tuning File
Qarray Bias :Use the Data in the Tuning File
Q1 Prerod Bias :Use the Data in the Tuning File
Q3 Prerod Bias :Use the Data in the Tuning File
--Segment 26 Event 26--
Acquisition Mode :MRM

Polarity :Positive
Start Time :10.808 min
End Time :13.808 min
Compound Name :TFAEEVNEAFR_light
Ch1 (Precursor m/z) 656.80 (Product m/z) 993.45 (Pause Time) 1.0 (Dwell Time) 5.0 (CE) -25.8
Ch2 (Precursor m/z) 656.80 (Product m/z) 864.40 (Pause Time) 1.0 (Dwell Time) 5.0 (CE) -25.8
Ch3 (Precursor m/z) 656.80 (Product m/z) 735.40 (Pause Time) 1.0 (Dwell Time) 5.0 (CE) -25.8
Ch4 (Precursor m/z) 656.80 (Product m/z) 636.30 (Pause Time) 1.0 (Dwell Time) 5.0 (CE) -25.8
Ch5 (Precursor m/z) 656.80 (Product m/z) 393.20 (Pause Time) 1.0 (Dwell Time) 5.0 (CE) -25.8
Event Time :0.030 sec
Q1 Resolution :Unit
Q3 Resolution :Unit
Micro Scan Width :0.00 u
Interface Volt. :4.00 kV
DUIS Corona Needle Volt. :4.50 kV
Pause Time / Dwell Time :Set Value of Each Channel (Pause Time & Dwell Time)
DL Bias :Use the Data in the Tuning File
Qarray Bias :Use the Data in the Tuning File
Q1 Prerod Bias :Use the Data in the Tuning File
Q3 Prerod Bias :Use the Data in the Tuning File
--Segment 27 Event 27--
Acquisition Mode :MRM
Polarity :Positive
Start Time :11.402 min
End Time :14.402 min
Compound Name :GTLFPMC[+57.0]GMNLAFDR_light
Ch1 (Precursor m/z) 577.25 (Product m/z) 735.40 (Pause Time) 1.0 (Dwell Time) 10.0 (CE) -20.5
Ch2 (Precursor m/z) 577.25 (Product m/z) 621.35 (Pause Time) 1.0 (Dwell Time) 10.0 (CE) -20.5
Ch3 (Precursor m/z) 577.25 (Product m/z) 508.25 (Pause Time) 1.0 (Dwell Time) 10.0 (CE) -20.5
Ch4 (Precursor m/z) 577.25 (Product m/z) 542.25 (Pause Time) 1.0 (Dwell Time) 10.0 (CE) -20.5
Ch5 (Precursor m/z) 577.25 (Product m/z) 311.15 (Pause Time) 1.0 (Dwell Time) 10.0 (CE) -20.5
Event Time :0.055 sec
Q1 Resolution :Unit
Q3 Resolution :Unit
Micro Scan Width :0.00 u
Interface Volt. :4.00 kV
DUIS Corona Needle Volt. :4.50 kV
Pause Time / Dwell Time :Set Value of Each Channel (Pause Time & Dwell Time)
DL Bias :Use the Data in the Tuning File
Qarray Bias :Use the Data in the Tuning File
Q1 Prerod Bias :Use the Data in the Tuning File
Q3 Prerod Bias :Use the Data in the Tuning File
--Segment 28 Event 28--
Acquisition Mode :MRM
Polarity :Positive
Start Time :11.482 min
End Time :14.482 min
Compound Name :IYLDISDEIK_light
Ch1 (Precursor m/z) 604.80 (Product m/z) 819.40 (Pause Time) 1.0 (Dwell Time) 4.0 (CE) -23.7
Ch2 (Precursor m/z) 604.80 (Product m/z) 704.40 (Pause Time) 1.0 (Dwell Time) 4.0 (CE) -23.7
Ch3 (Precursor m/z) 604.80 (Product m/z) 591.30 (Pause Time) 1.0 (Dwell Time) 4.0 (CE) -23.7
Ch4 (Precursor m/z) 604.80 (Product m/z) 504.25 (Pause Time) 1.0 (Dwell Time) 4.0 (CE) -23.7
Ch5 (Precursor m/z) 604.80 (Product m/z) 389.25 (Pause Time) 1.0 (Dwell Time) 4.0 (CE) -23.7
Ch6 (Precursor m/z) 604.80 (Product m/z) 260.20 (Pause Time) 1.0 (Dwell Time) 4.0 (CE) -23.7
Event Time :0.030 sec
Q1 Resolution :Unit
Q3 Resolution :Unit
Micro Scan Width :0.00 u
Interface Volt. :4.00 kV
DUIS Corona Needle Volt. :4.50 kV
Pause Time / Dwell Time :Set Value of Each Channel (Pause Time & Dwell Time)
DL Bias :Use the Data in the Tuning File
Qarray Bias :Use the Data in the Tuning File
Q1 Prerod Bias :Use the Data in the Tuning File
Q3 Prerod Bias :Use the Data in the Tuning File
--Segment 29 Event 29--
Acquisition Mode :MRM
Polarity :Positive
Start Time :11.848 min
End Time :14.848 min
Compound Name :IIGVTTLDVVR_light
Ch1 (Precursor m/z) 593.35 (Product m/z) 803.45 (Pause Time) 1.0 (Dwell Time) 6.0 (CE) -23.2
Ch2 (Precursor m/z) 593.35 (Product m/z) 702.40 (Pause Time) 1.0 (Dwell Time) 6.0 (CE) -23.2
Ch3 (Precursor m/z) 593.35 (Product m/z) 601.35 (Pause Time) 1.0 (Dwell Time) 6.0 (CE) -23.2
Ch4 (Precursor m/z) 593.35 (Product m/z) 488.30 (Pause Time) 1.0 (Dwell Time) 6.0 (CE) -23.2
Ch5 (Precursor m/z) 593.35 (Product m/z) 373.25 (Pause Time) 1.0 (Dwell Time) 6.0 (CE) -23.2
Event Time :0.035 sec
Q1 Resolution :Unit
Q3 Resolution :Unit
Micro Scan Width :0.00 u
Interface Volt. :4.00 kV

DUIS Corona Needle Volt. :4.50 kV
Pause Time / Dwell Time :Set Value of Each Channel (Pause Time & Dwell Time)
DL Bias :Use the Data in the Tuning File
Qarray Bias :Use the Data in the Tuning File
Q1 Prerod Bias :Use the Data in the Tuning File
Q3 Prerod Bias :Use the Data in the Tuning File
--Segment 30 Event 30--
Acquisition Mode :MRM
Polarity :Positive
Start Time :11.979 min
End Time :14.979 min
Compound Name :SYELPDGQVITIGNER_light
Ch1 (Precursor m/z) 895.95 (Product m/z) 475.25 (Pause Time) 1.0 (Dwell Time) 10.0 (CE) -35.3
Ch2 (Precursor m/z) 895.95 (Product m/z) 418.20 (Pause Time) 1.0 (Dwell Time) 10.0 (CE) -35.3
Ch3 (Precursor m/z) 895.95 (Product m/z) 515.30 (Pause Time) 1.0 (Dwell Time) 10.0 (CE) -35.3
Ch4 (Precursor m/z) 895.95 (Product m/z) 451.25 (Pause Time) 1.0 (Dwell Time) 10.0 (CE) -35.3
Ch5 (Precursor m/z) 895.95 (Product m/z) 345.20 (Pause Time) 1.0 (Dwell Time) 10.0 (CE) -35.3
Event Time :0.055 sec
Q1 Resolution :Unit
Q3 Resolution :Unit
Micro Scan Width :0.00 u
Interface Volt. :4.00 kV
DUIS Corona Needle Volt. :4.50 kV
Pause Time / Dwell Time :Set Value of Each Channel (Pause Time & Dwell Time)
DL Bias :Use the Data in the Tuning File
Qarray Bias :Use the Data in the Tuning File
Q1 Prerod Bias :Use the Data in the Tuning File
Q3 Prerod Bias :Use the Data in the Tuning File
--Segment 31 Event 31--
Acquisition Mode :MRM
Polarity :Positive
Start Time :11.985 min
End Time :14.985 min
Compound Name :SYELPDGQVITIGNER_light
Ch1 (Precursor m/z) 597.65 (Product m/z) 475.25 (Pause Time) 1.0 (Dwell Time) 11.0 (CE) -21.3
Ch2 (Precursor m/z) 597.65 (Product m/z) 706.40 (Pause Time) 1.0 (Dwell Time) 11.0 (CE) -21.3
Ch3 (Precursor m/z) 597.65 (Product m/z) 515.30 (Pause Time) 1.0 (Dwell Time) 11.0 (CE) -21.3
Event Time :0.036 sec
Q1 Resolution :Unit
Q3 Resolution :Unit
Micro Scan Width :0.00 u
Interface Volt. :4.00 kV
DUIS Corona Needle Volt. :4.50 kV
Pause Time / Dwell Time :Set Value of Each Channel (Pause Time & Dwell Time)
DL Bias :Use the Data in the Tuning File
Qarray Bias :Use the Data in the Tuning File
Q1 Prerod Bias :Use the Data in the Tuning File
Q3 Prerod Bias :Use the Data in the Tuning File
--Segment 32 Event 32--
Acquisition Mode :MRM
Polarity :Positive
Start Time :12.285 min
End Time :15.285 min
Compound Name :LDVAIDGADEVDPNLDVVK_light
Ch1 (Precursor m/z) 666.35 (Product m/z) 998.55 (Pause Time) 1.0 (Dwell Time) 10.0 (CE) -23.8
Ch2 (Precursor m/z) 666.35 (Product m/z) 899.50 (Pause Time) 1.0 (Dwell Time) 10.0 (CE) -23.8
Ch3 (Precursor m/z) 666.35 (Product m/z) 784.45 (Pause Time) 1.0 (Dwell Time) 10.0 (CE) -23.8
Ch4 (Precursor m/z) 666.35 (Product m/z) 345.25 (Pause Time) 1.0 (Dwell Time) 10.0 (CE) -23.8
Ch5 (Precursor m/z) 666.35 (Product m/z) 450.25 (Pause Time) 1.0 (Dwell Time) 10.0 (CE) -23.8
Ch6 (Precursor m/z) 666.35 (Product m/z) 392.75 (Pause Time) 1.0 (Dwell Time) 10.0 (CE) -23.8
Event Time :0.066 sec
Q1 Resolution :Unit
Q3 Resolution :Unit
Micro Scan Width :0.00 u
Interface Volt. :4.00 kV
DUIS Corona Needle Volt. :4.50 kV
Pause Time / Dwell Time :Set Value of Each Channel (Pause Time & Dwell Time)
DL Bias :Use the Data in the Tuning File
Qarray Bias :Use the Data in the Tuning File
Q1 Prerod Bias :Use the Data in the Tuning File
Q3 Prerod Bias :Use the Data in the Tuning File
--Segment 33 Event 33--
Acquisition Mode :MRM
Polarity :Positive
Start Time :12.362 min
End Time :15.362 min
Compound Name :DLGGYDKFVEAFK_light
Ch1 (Precursor m/z) 496.90 (Product m/z) 740.40 (Pause Time) 1.0 (Dwell Time) 6.0 (CE) -17.5
Ch2 (Precursor m/z) 496.90 (Product m/z) 593.35 (Pause Time) 1.0 (Dwell Time) 6.0 (CE) -17.5
Ch3 (Precursor m/z) 496.90 (Product m/z) 494.25 (Pause Time) 1.0 (Dwell Time) 6.0 (CE) -17.5
Ch4 (Precursor m/z) 496.90 (Product m/z) 365.20 (Pause Time) 1.0 (Dwell Time) 6.0 (CE) -17.5

Ch5 (Precursor m/z) 496.90 (Product m/z) 602.30 (Pause Time) 1.0 (Dwell Time) 6.0 (CE) -17.5
Event Time :0.035 sec
Q1 Resolution :Unit
Q3 Resolution :Unit
Micro Scan Width :0.00 u
Interface Volt. :4.00 kV
DUIS Corona Needle Volt. :4.50 kV
Pause Time / Dwell Time :Set Value of Each Channel (Pause Time & Dwell Time)
DL Bias :Use the Data in the Tuning File
Qarray Bias :Use the Data in the Tuning File
Q1 Prerod Bias :Use the Data in the Tuning File
Q3 Prerod Bias :Use the Data in the Tuning File
--Segment 34 Event 34--
Acquisition Mode :MRM
Polarity :Positive
Start Time :12.561 min
End Time :15.561 min
Compound Name :VVVAYEPVWAIGTGK_light
Ch1 (Precursor m/z) 530.30 (Product m/z) 369.25 (Pause Time) 1.0 (Dwell Time) 6.0 (CE) -18.8
Ch2 (Precursor m/z) 530.30 (Product m/z) 661.35 (Pause Time) 1.0 (Dwell Time) 6.0 (CE) -18.8
Ch3 (Precursor m/z) 530.30 (Product m/z) 857.50 (Pause Time) 1.0 (Dwell Time) 6.0 (CE) -18.8
Ch4 (Precursor m/z) 530.30 (Product m/z) 331.20 (Pause Time) 1.0 (Dwell Time) 6.0 (CE) -18.8
Ch5 (Precursor m/z) 530.30 (Product m/z) 379.70 (Pause Time) 1.0 (Dwell Time) 6.0 (CE) -18.8
Event Time :0.035 sec
Q1 Resolution :Unit
Q3 Resolution :Unit
Micro Scan Width :0.00 u
Interface Volt. :4.00 kV
DUIS Corona Needle Volt. :4.50 kV
Pause Time / Dwell Time :Set Value of Each Channel (Pause Time & Dwell Time)
DL Bias :Use the Data in the Tuning File
Qarray Bias :Use the Data in the Tuning File
Q1 Prerod Bias :Use the Data in the Tuning File
Q3 Prerod Bias :Use the Data in the Tuning File
--Segment 35 Event 35--
Acquisition Mode :MRM
Polarity :Positive
Start Time :13.364 min
End Time :16.364 min
Compound Name :AAGATQFGSGWAWLSVNK_light
Ch1 (Precursor m/z) 925.95 (Product m/z) 1204.60 (Pause Time) 1.0 (Dwell Time) 10.0 (CE) -36.5
Ch2 (Precursor m/z) 925.95 (Product m/z) 817.45 (Pause Time) 1.0 (Dwell Time) 10.0 (CE) -36.5
Ch3 (Precursor m/z) 925.95 (Product m/z) 560.35 (Pause Time) 1.0 (Dwell Time) 10.0 (CE) -36.5
Ch4 (Precursor m/z) 925.95 (Product m/z) 447.25 (Pause Time) 1.0 (Dwell Time) 10.0 (CE) -36.5
Ch5 (Precursor m/z) 925.95 (Product m/z) 360.20 (Pause Time) 1.0 (Dwell Time) 10.0 (CE) -36.5
Event Time :0.055 sec
Q1 Resolution :Unit
Q3 Resolution :Unit
Micro Scan Width :0.00 u
Interface Volt. :4.00 kV
DUIS Corona Needle Volt. :4.50 kV
Pause Time / Dwell Time :Set Value of Each Channel (Pause Time & Dwell Time)
DL Bias :Use the Data in the Tuning File
Qarray Bias :Use the Data in the Tuning File
Q1 Prerod Bias :Use the Data in the Tuning File
Q3 Prerod Bias :Use the Data in the Tuning File
--Segment 36 Event 36--
Acquisition Mode :MRM
Polarity :Positive
Start Time :14.423 min
End Time :17.423 min
Compound Name :DLESLSIEEVMLK_light
Ch1 (Precursor m/z) 753.40 (Product m/z) 1148.60 (Pause Time) 1.0 (Dwell Time) 10.0 (CE) -29.6
Ch2 (Precursor m/z) 753.40 (Product m/z) 948.50 (Pause Time) 1.0 (Dwell Time) 10.0 (CE) -29.6
Ch3 (Precursor m/z) 753.40 (Product m/z) 861.45 (Pause Time) 1.0 (Dwell Time) 10.0 (CE) -29.6
Ch4 (Precursor m/z) 753.40 (Product m/z) 748.40 (Pause Time) 1.0 (Dwell Time) 10.0 (CE) -29.6
Ch5 (Precursor m/z) 753.40 (Product m/z) 391.25 (Pause Time) 1.0 (Dwell Time) 10.0 (CE) -29.6
Event Time :0.055 sec
Q1 Resolution :Unit
Q3 Resolution :Unit
Micro Scan Width :0.00 u
Interface Volt. :4.00 kV
DUIS Corona Needle Volt. :4.50 kV
Pause Time / Dwell Time :Set Value of Each Channel (Pause Time & Dwell Time)
DL Bias :Use the Data in the Tuning File
Qarray Bias :Use the Data in the Tuning File
Q1 Prerod Bias :Use the Data in the Tuning File
Q3 Prerod Bias :Use the Data in the Tuning File
--Segment 37 Event 37--
Acquisition Mode :MRM
Polarity :Positive

Start Time :15.039 min
End Time :18.039 min
Compound Name :VAVLGAAGGIGQPLSLLMK_light

Ch1	(Precursor m/z) 599.00	(Product m/z) 986.55	(Pause Time) 1.0	(Dwell Time) 20.0	(CE) -21.3
Ch2	(Precursor m/z) 599.00	(Product m/z) 801.50	(Pause Time) 1.0	(Dwell Time) 20.0	(CE) -21.3
Ch3	(Precursor m/z) 599.00	(Product m/z) 591.35	(Pause Time) 1.0	(Dwell Time) 20.0	(CE) -21.3
Ch4	(Precursor m/z) 599.00	(Product m/z) 391.25	(Pause Time) 1.0	(Dwell Time) 20.0	(CE) -21.3
Ch5	(Precursor m/z) 599.00	(Product m/z) 401.25	(Pause Time) 1.0	(Dwell Time) 20.0	(CE) -21.3

Event Time :0.105 sec
Q1 Resolution :Unit
Q3 Resolution :Unit
Micro Scan Width :0.00 u
Interface Volt. :4.00 kV
DUIS Corona Needle Volt. :4.50 kV
Pause Time / Dwell Time :Set Value of Each Channel (Pause Time & Dwell Time)
DL Bias :Use the Data in the Tuning File
Qarray Bias :Use the Data in the Tuning File
Q1 Prerod Bias :Use the Data in the Tuning File
Q3 Prerod Bias :Use the Data in the Tuning File
--Segment 38 Event 38--
Acquisition Mode :MRM
Polarity :Positive
Start Time :15.132 min
End Time :18.132 min
Compound Name :VAVLGAAGGIGQPLSLLMK_light

Ch1	(Precursor m/z) 898.05	(Product m/z) 1213.70	(Pause Time) 1.0	(Dwell Time) 20.0	(CE) -35.4
Ch2	(Precursor m/z) 898.05	(Product m/z) 986.55	(Pause Time) 1.0	(Dwell Time) 20.0	(CE) -35.4
Ch3	(Precursor m/z) 898.05	(Product m/z) 801.50	(Pause Time) 1.0	(Dwell Time) 20.0	(CE) -35.4
Ch4	(Precursor m/z) 898.05	(Product m/z) 591.35	(Pause Time) 1.0	(Dwell Time) 20.0	(CE) -35.4
Ch5	(Precursor m/z) 898.05	(Product m/z) 391.25	(Pause Time) 1.0	(Dwell Time) 20.0	(CE) -35.4

Event Time :0.105 sec
Q1 Resolution :Unit
Q3 Resolution :Unit
Micro Scan Width :0.00 u
Interface Volt. :4.00 kV
DUIS Corona Needle Volt. :4.50 kV
Pause Time / Dwell Time :Set Value of Each Channel (Pause Time & Dwell Time)
DL Bias :Use the Data in the Tuning File
Qarray Bias :Use the Data in the Tuning File
Q1 Prerod Bias :Use the Data in the Tuning File
Q3 Prerod Bias :Use the Data in the Tuning File
--Segment 39 Event 39--
Acquisition Mode :MRM
Polarity :Positive
Start Time :17.337 min
End Time :20.337 min
Compound Name :LIEYATQEGLVLILVC[+57.0]ASGGAR_light

Ch1	(Precursor m/z) 778.40	(Product m/z) 678.30	(Pause Time) 1.0	(Dwell Time) 40.0	(CE) -28.0
Ch2	(Precursor m/z) 778.40	(Product m/z) 447.25	(Pause Time) 1.0	(Dwell Time) 40.0	(CE) -28.0
Ch3	(Precursor m/z) 778.40	(Product m/z) 303.20	(Pause Time) 1.0	(Dwell Time) 40.0	(CE) -28.0
Ch4	(Precursor m/z) 778.40	(Product m/z) 664.90	(Pause Time) 1.0	(Dwell Time) 40.0	(CE) -28.0
Ch5	(Precursor m/z) 778.40	(Product m/z) 608.35	(Pause Time) 1.0	(Dwell Time) 40.0	(CE) -28.0

Event Time :0.205 sec
Q1 Resolution :Unit
Q3 Resolution :Unit
Micro Scan Width :0.00 u
Interface Volt. :4.00 kV
DUIS Corona Needle Volt. :4.50 kV
Pause Time / Dwell Time :Set Value of Each Channel (Pause Time & Dwell Time)
DL Bias :Use the Data in the Tuning File
Qarray Bias :Use the Data in the Tuning File
Q1 Prerod Bias :Use the Data in the Tuning File
Q3 Prerod Bias :Use the Data in the Tuning File
--Segment 40 Event 40--
Acquisition Mode :MRM
Polarity :Positive
Start Time :17.360 min
End Time :20.360 min
Compound Name :ILFEVAPLALLVEK_light

Ch1	(Precursor m/z) 778.00	(Product m/z) 1052.65	(Pause Time) 1.0	(Dwell Time) 30.0	(CE) -30.6
Ch2	(Precursor m/z) 778.00	(Product m/z) 953.60	(Pause Time) 1.0	(Dwell Time) 30.0	(CE) -30.6
Ch3	(Precursor m/z) 778.00	(Product m/z) 882.55	(Pause Time) 1.0	(Dwell Time) 30.0	(CE) -30.6
Ch4	(Precursor m/z) 778.00	(Product m/z) 601.40	(Pause Time) 1.0	(Dwell Time) 30.0	(CE) -30.6
Ch5	(Precursor m/z) 778.00	(Product m/z) 375.20	(Pause Time) 1.0	(Dwell Time) 30.0	(CE) -30.6

Event Time :0.155 sec
Q1 Resolution :Unit
Q3 Resolution :Unit
Micro Scan Width :0.00 u
Interface Volt. :4.00 kV
DUIS Corona Needle Volt. :4.50 kV
Pause Time / Dwell Time :Set Value of Each Channel (Pause Time & Dwell Time)

DL Bias	:Use the Data in the Tuning File
Qarray Bias	:Use the Data in the Tuning File
Q1 Prerod Bias	:Use the Data in the Tuning File
Q3 Prerod Bias	:Use the Data in the Tuning File
